# Associations between people experiencing homelessness (PEH) and neurodegenerative disorders (NDDs): A systematic review and meta-analysis

**DOI:** 10.1371/journal.pone.0312117

**Published:** 2024-10-22

**Authors:** Pengfei Fu, Vijay Mago, Rebecca Schiff, Bonnie Krysowaty

**Affiliations:** 1 School of Health Policy and Management, Faculty of Health, York University, Toronto, Ontario, Canada; 2 University of Lethbridge, Lethbridge, Alberta, Canada; 3 Lakehead Social Planning Council, Victoriaville Centre, Thunder Bay, Ontario, Canada; Global Health Neurology Lab / NSW Brain Clot Bank, NSW Health Pathology / Liverpool Hospital and South West Sydney Local Health District / Neurovascular Imaging Lab, Clinical Sciences Stream, Ingham Institute, AUSTRALIA

## Abstract

**Background:**

Homelessness represents a widespread social issue globally, yet the risk of neurodegenerative diseases (NDDs) associated with people experiencing homelessness (PEH) has not received sufficient attention. Therefore, this study aimed to explore the risk of NDDs among PEH and its variation across countries and regions through meta-analysis and systematic review.

**Methods:**

Searching from databases such as PubMed and Web of Science, relevant research articles on PEH and NDDs were identified. After multiple screening, eight articles were selected for meta-analysis. Statistical methods and models were used to evaluate the association between PEH and NDDs, stratified by disease type and country.

**Results:**

We found that PEH had a 51% higher risk of NDDs (OR = 1.51 (95% CI: 1.21, 1.89)) compared with those with stable housing. Specifically, PEH had a significantly higher risk of developing multiple sclerosis (OR = 4.64 (95% CI: 1.96, 10.98)). Alzheimer’s disease and related dementias (ADRD) (OR = 1.93 (95% CI: 1.34, 2.77)), dementia (OR = 1.69 (95% CI: 1.26, 2.27)), and cognitive impairment (OR = 1.07 (95% CI: 0.98, 1.16)) were all at higher risk. Furthermore, country and regional differences were observed, with countries such as Iran (OR = 4.64 (95% CI: 1.96, 10.98)), the Netherlands (OR = 2.14 (95% CI: 1.23, 3.73)), the United States (OR = 1.66 (95% CI: 1.25, 2.22)), and Canada (OR = 1.06 (95% CI: 1.01, 1.10)) showing a higher risk of NDDs among the PEH.

**Conclusions:**

The study emphasizes the significant NDD risks among PEH, providing novel perspectives on this issue and shedding light on national disparities influenced by variations in healthcare systems and social environments. This will be beneficial for academia and government to prioritize the health of PEH with NDDs, aiming to mitigate disease incidence and economic burdens while preserving social stability and upholding basic human rights.

## Introduction

Homelessness presents a global challenge with social, economic, and health implications. It extends beyond associations with economically disadvantaged areas and emerges as a significant concern in developed countries as well. According to research published in *The Lancet*, the European Union alone records a nightly count exceeding 400,000 people experiencing homelessness (PEH) [1]. In the United States, over 560,000 people experience homelessness each night. Over the past decade, the number of PEH has increased by 4%. Cardiovascular disease is the leading cause of death among PEH, with a mortality rate three times higher than that of the general population [2]. Centralized housing policies in the Netherlands have been criticized for their inability to address housing shortages and support basic human rights [3]. Even in Denmark, Europe’s largest housing priority system, only 10% of PEH are adequately served [3]. PEH are also of great concern in Canada, with research pointing to the importance of PEH not only in terms of its economic impact but also on health status, including significant impacts on mental health issues related to basic human needs, psycho-emotional needs, and the social determinants of mental health [4]. Studying the risk factors associated with PEH is important for social stability and well-being.

Neurodegenerative diseases (NDDs) are disabling conditions that cause progressive motor and cognitive impairments, including Alzheimer’s disease (AD, the most common cause of dementia), other forms of dementia, multiple sclerosis (MS), Parkinson’s disease, Huntington’s chorea, and other disorders [5]. NDDs place a heavy social and economic burden on countries worldwide [5]. For instance, AD, renowned as one of this century’s most lethal and incapacitating ailments, is a formidable global challenge [6]. The prevalence of Alzheimer’s disease is rapidly increasing. In 2018 alone, there were approximately 50 million people with dementia worldwide, and this number is expected to triple by 2050 [6]. In Europe, for example, the prevalence of dementia is expected to double by 2050. Simultaneously, two-thirds of people with dementia live in low- and middle-income countries [6].

In recent years, emerging evidence suggests that the prevalence of NDDs is increasing among PEH, particularly those aged 18–49 and among veterans [7, 8]. This suggests that the onset of NDDs in PEH is occurring at an earlier age than the typical age of onset, posing significant challenges to social stability, burden, and basic human rights. However, the relationship between PEH and NDDs remains inadequately understood. A small number of articles have discussed this relationship, but they lacked quantitative results or were limited to discussing only one type of NDD. For example, one study suggested a role for NDDs in promoting homelessness in older adults but did not present quantifiable risks (e.g., prevalence rate or OR) [9]. Another review on homelessness and ADRD suggested that homelessness may be both a risk and a consequence of ADRD but again did not provide quantifiable risk indicators to emphasize the strength of this association [7]. Studies that provided ORs mentioned that unmarried young adults with an ADRD diagnosis were associated with a high risk of homelessness, but they only discussed one disease and provided results from one study [10]. Therefore, research on this association with quantified risk estimates can provide an important basis for mitigating the health risks of PEH and shorten the distance of the current studies.

This study aims to examine the association between PEH and NDDs through a systematic review and meta-analysis of the existing studies. We aimed to quantify the severity of NDDs’ risk (e.g., AD, dementia, MS, and cognitive impairment) in the PEH and explore potential differences across countries. Through comparative analysis of various NDD risks among PEH across different countries, this study aims to discuss the socioeconomic factors, healthcare system, and environmental exposures underlying this adverse association. The quantitative results can guide targeted interventions and policy formulations to mitigate NDD risks among PEH, thereby advancing social equity. It stands to furnish support for subsequent investigations and facilitate the formulation of comprehensive public health initiatives tailored to address the multifaceted challenges posed by the intersection of PEH and NDDs on a global scale.

## Material and methods

### Search strategy and selection criteria

We utilized PICO’s methodology to systematically identify our research questions and key elements. The participants were individuals with NDD. NDDs served as both the intervention/exposure and the outcome of interest. Participants in the included studies had at least one NDD. Comparisons were made by evaluating the difference in risk of NDDs between PEH and non-PEH, using meta-regression modeling. The outcome of interest was the risk of each NDD in PEH, with results expressed as odds ratios (OR) and 95% confidence intervals (CI).

**Fig 1:**
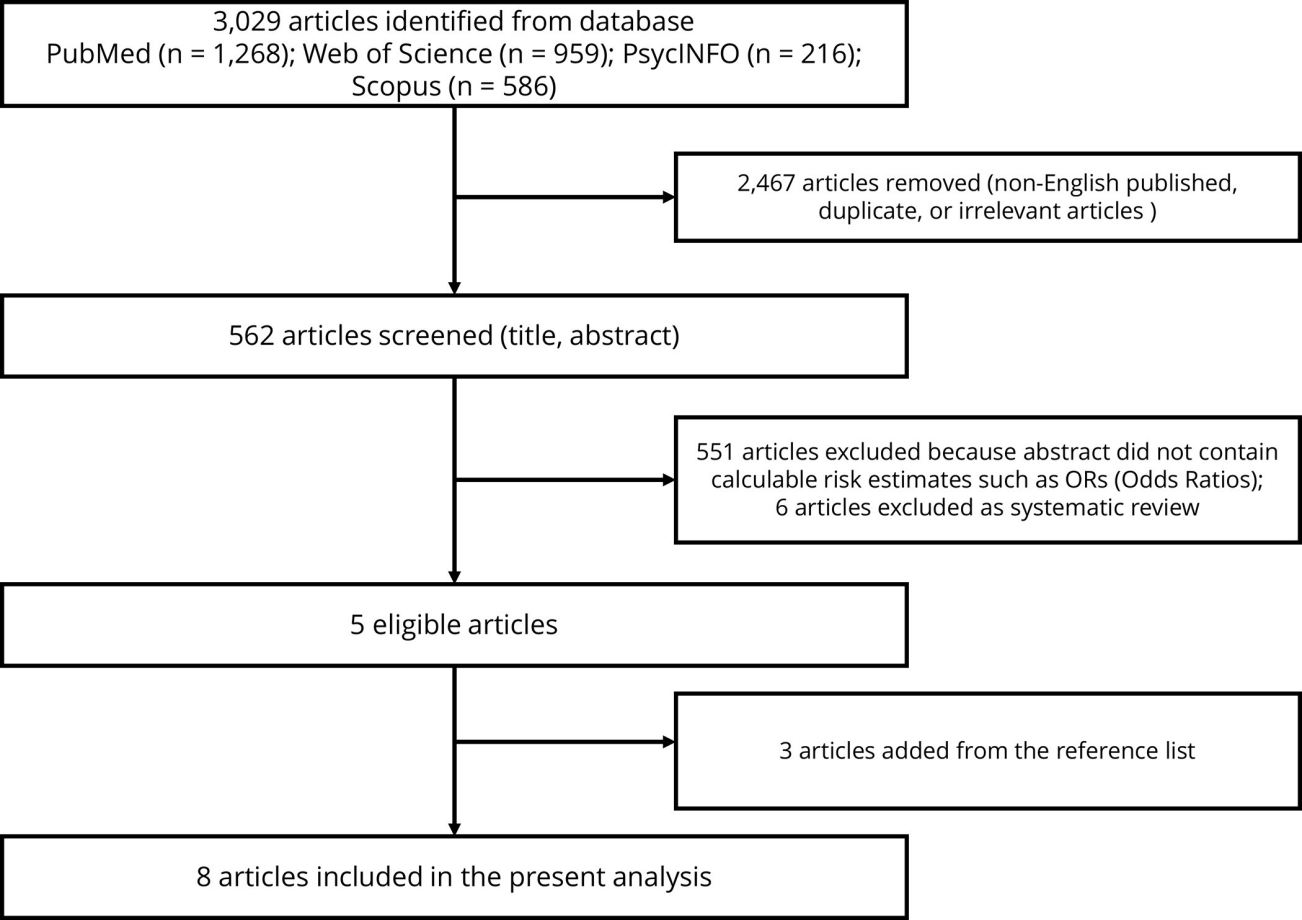
Flow diagram.

We examined the association between people experiencing homelessness (PEH) and neurodegenerative disorders (NDDs) using PubMed, Web of Science, PsycINFO and Scopus (Fig 1). Search terms included “homeless” (without limitation on the duration of homelessness) and common NDDs such as “dementia,” “Alzheimer’s disease,” “Parkinson’s disease,” and “neurodegenerative disease.” Detailed search terms are provided in S1 Table. Articles included in this analysis had to be published in English and provide calculable risk estimates and their 95% CIs, such as ORs, relative risks (RRs), hazard ratios (HRs), regression coefficients (βs), or percentage changes (%). These articles needed to demonstrate the impact of PEH as a risk factor for NDDs. Study design (e.g., whether it was only peer-reviewed articles or cohort studies) was not used as a restriction or exclusionary criteria as we wanted to collect as much data as possible in order to draw a more comprehensive analysis. We reviewed the tables and content of the articles and excluded duplicates or irrelevant articles. To ensure comprehensiveness, we supplemented the included articles with literature lists from relevant studies and systematic reviews. Study selection was conducted independently by the author (PF). Any disagreements between the reviewers were resolved through consultation with the other authors (VM, RS, BK).

### Data analysis

A modified standardized approach was used to summarize the included studies [11–13]. This approach documented the first author, year of publication, country, disease type (e.g., Alzheimer’s disease, dementia, or multiple sclerosis), study design (e.g., case-control, time-series, cross-sectional, or cohort study), age range, risk estimates, and their 95% CIs (Table 1). If specific data were not reported in the original sources, they were marked as N.A. Age ranges were recorded as provided; if an age range was not specified but an average age was, the average age was noted. The authors independently collected information and reviewed it to ensure the data collection process was thoroughly tested multiple times. To enhance comprehensiveness, we included as much data as possible from each source, which may have resulted in some studies appearing multiple times in the results. Studies were categorized by disease type. For AD, ADRD, and dementia, we first combined them to calculate the overall OR, followed by a subgroup analysis to calculate the combined ORs for dementia, including all three types. We calculated ORs for meta-analysis for studies that did not directly report ORs but provided calculable risk estimates or 2x2 contingency tables. For data provided with a 2x2 contingency table, we used the csi command in Stata software to convert the OR [14]. Data provided with a β value and a 95% CI were converted using the formula: OR = exp(β).

**Table 1:** Descriptive data of the included studies.

Study	Year	NDD Types	Country	Study Design	Population	Period	Age	Model Adjustment	Title
Jutkowitz et al. [8]	2021	ADRD	United States	Cross-sectional study	6580126	2018	All ages	Used logistic regression and calculated the unadjusted (just controls for age) and adjusted (controls for all demographics and comorbidities) relative risk of the association between and comorbidities) relative risk of the association between and comorbidities) relative risk of the association between housing status and having an AD/ADRD diagnosis.	Prevalence of Alzheimer’s disease and related dementias among veterans experiencing housing insecurity
Roncarati JS et al. [15]	2024	ADRD	United States	Cohort study	88811	2011–2019	63.5	Estimated the unadjusted and adjusted rate of being diagnosed with ADRD using cox proportional hazard models on the matched cohort. The adjusted model, all the independent variables obtained from the Corporate Data Warehouse (age, race, not married, combat veteran, rurality, comorbidities).	Risk of dementia among veterans experiencing homelessness and housing instability
Keigher et al. [16]	1992	Dementia	United States	Cross-sectional study	475	1987–1988	≥ 60	No confounders for adjustment.	Housing emergencies and the etiology of homelessness among the urban elderly
Jutkowitz et al. [17]	2019	Dementia	United States	Cross-sectional study	114013	2010–2017	54.5	Estimated the adjusted relative risk (ARR) of each condition/nursing home measure in homeless veterans compared to stably housed veterans and in at-risk veterans compared to stably housed veterans.	Homeless Veterans in Nursing Homes: Care for Complex Medical, Substance Use, and Social Needs
Ye et al. [18]	2019	Memory loss	United States	Cross-sectional study	64	2016–2018	≥ 50	No confounders for adjustment.	Health Care Needs of Homeless Older Adults: Examining the Needs of a Senior Center Cohort
Stergiopoulos et al. [19]	2019	Cognitive Impairment	Canada	N.A.	902	2009–2011	≥ 18	Multivariable linear regression models examined the associations between all NP outcomes at 24-month and housing stability, adjusting for the outcome score at 6 months.	Housing Stability and Neurocognitive Functioning in Homeless Adults With Mental Illness: A Subgroup Analysis of the At Home/Chez Soi Study
Van Straaten et al. [20]	2017	Cognitive Impairment	Netherlands	Cohort study	513	2011	≥ 18	Repeated-measures ANCOVA adjusted for age and gender.	Self-reported care needs of Dutch homeless people with and without a suspected intellectual disability: a 1.5-year follow-up study
Abdollahpour et al. [21]	2018	Multiple Sclerosis	Iran	Case-control study	1604	2013–2015	≥ 13	Logistic regression model was applied to estimate the odds ratios (95% CI) adjusted for physical activity, age, gender, tobacco smoking, waterpipe smoking and passive smoking.	Stress-full life events and multiple sclerosis: A population-based incident case-control study

To assess the quality of the studies included in the analysis, we used a scoring checklist adapted from the Agency for Healthcare Research and Quality and our previous work [11, 22]. Each study would receive a score (out of ten) if it met any of the criteria, and then the average score would be calculated. The scoring scheme can be found in the S2 Table.

Sensitivity analyses were performed using Stata’s *metaninf* module (S2 Fig). This module removed the included data individually to assess the impact of each data on the overall results. Heterogeneity analysis was reflected in I-squared values, which also determined the study model: if the I-squared value was less than 50%, a fixed model was selected; if it was greater, a random model was selected [12, 23]. Begg’s tests were used to detect publication bias (S1 Fig). Modeling for this study was performed using Stata 17.0 software (StataCorp LLC). This meta-analysis was not registered [12, 24]. An ethics statement was not required for this work.

## Results

A total of 3,029 articles from four databases were screened (Fig 1) (PubMed (n = 1,268); Web of Science (n = 959); PsycINFO (n = 216); Scopus (n = 586)). After an initial screening of 2,467 articles published in non-English languages, duplicates, or irrelevant articles, 562 articles were further screened. Of these, 557 articles were excluded, including 551 that did not contain calculable data, and six that were systematic reviews. We added three articles by reviewing the bibliography and finally included eight articles for meta-analysis [8, 15–21] (Table 1). Most of the studies were of high quality (mean score = 7.5) in the quality assessment, with no articles scoring less than five, indicating the high quality of the included data. No significant bias or its source was detected by Begg’s asymmetry test and the I^2^ test (S1 Fig). Sensitivity analysis results were similar to those of the original analysis, reflecting the robustness of the analysis results.

Our study retrieved articles published before July 24, 2024. The earliest study was published in 1992, and the latest in 2024. Beyond the overall OR, we stratified more comprehensive results by subgroup analysis. We first stratified by different types of NDDs, with 2 articles on ADRD, 5 articles on dementia (including ADRD and unspecified subtypes of dementia), 2 articles on cognitive impairment, and 1 article on MS. We then stratified by country, with 5 articles from the United States and 1 each from Canada, Netherlands, and Iran. The total number of studies may add up to more than 8 because some articles may address multiple disease types, countries, or report multiple outcomes.

**Fig 2:**
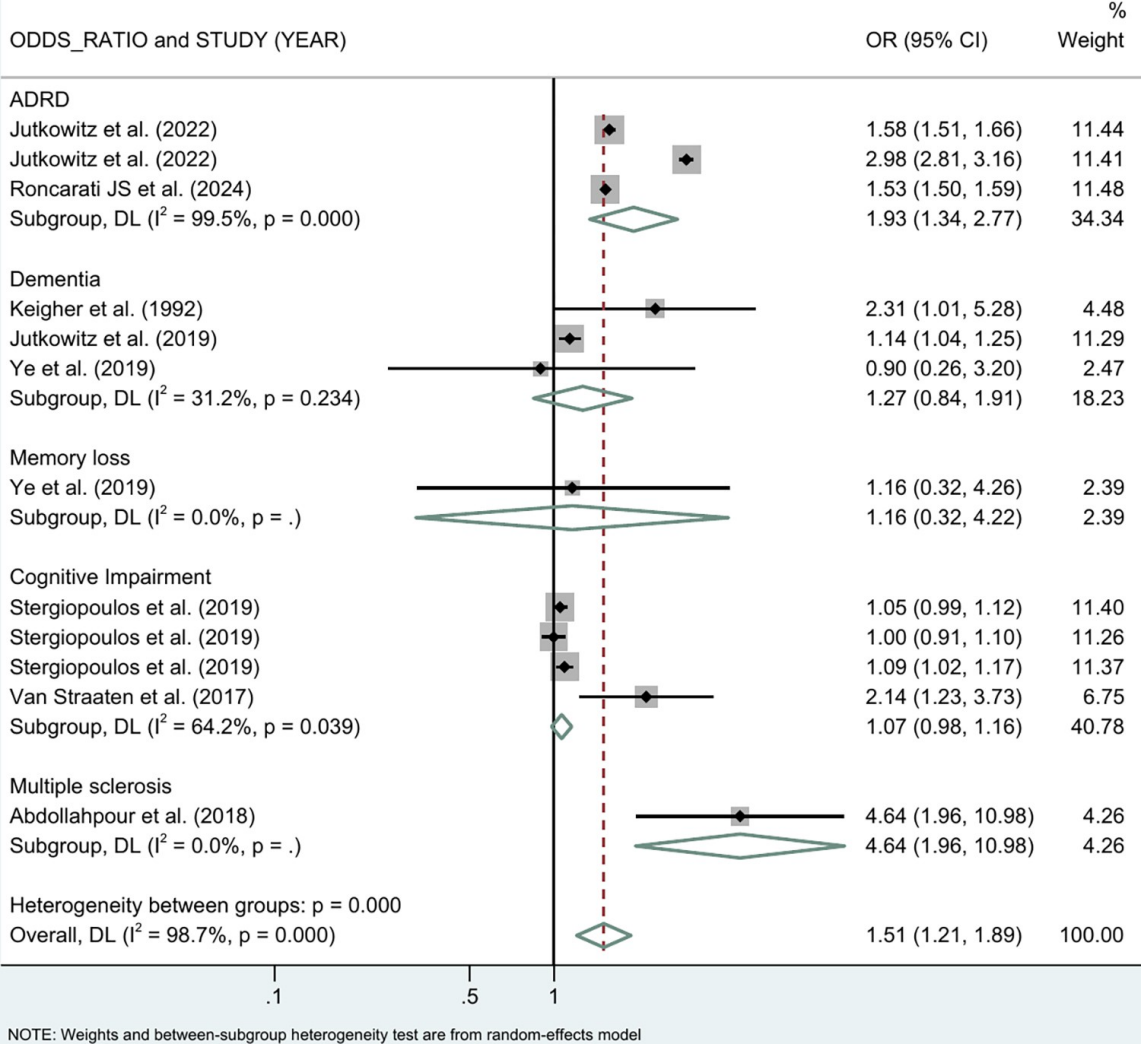
Meta-analysis of the association between PEH and NDDs.

**Fig 3:**
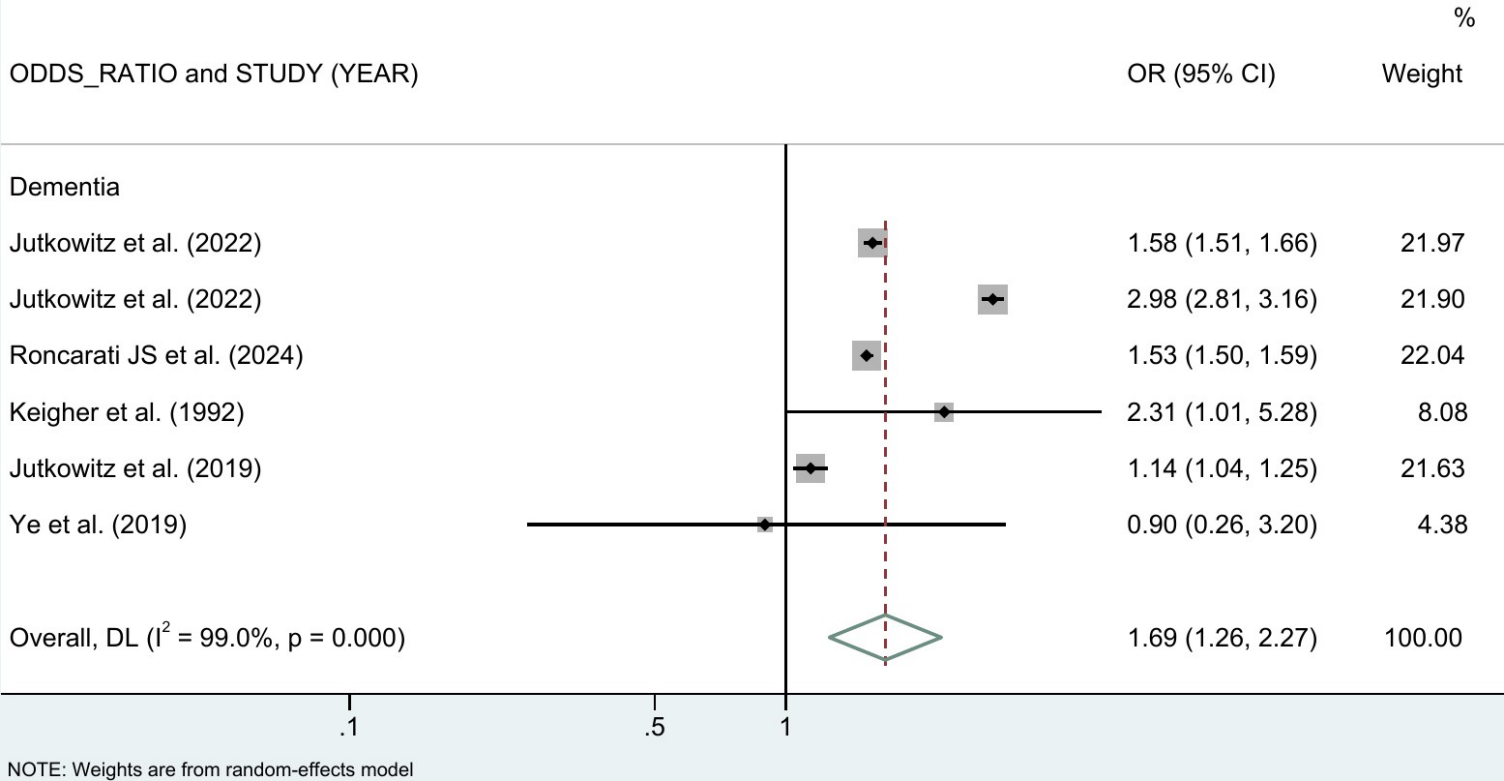
Meta-analysis of the association between PEH and NDDs stratified by dementia.

Fig 2 shows the major outcomes of different NDDs by meta-analysis results. Overall, the results showed that the risk of NDDs was 51% higher among PEH compared to those with stable housing (OR = 1.51 (95% CI: 1.21, 1.89)). The risk of ADRD was increased by 93% in PEH (OR = 1.93 (95% CI: 1.34, 2.77)), while the risk of dementia was increased by 69% when all dementia subtypes were combined (OR = 1.69 (95% CI: 1.26, 2.27)) (Fig 3). PEH also had a 16% increased risk of memory loss, a major symptom of dementia, and 7% increased risk of cognitive impairment (OR = 1.07 (95% CI: 0.98, 1.16)) (Fig 2). Although the data included were smaller, MS also showed an increased risk of PEH (OR = 4.64 (95% CI: 1.96, 10.98)).

**Fig 4:**
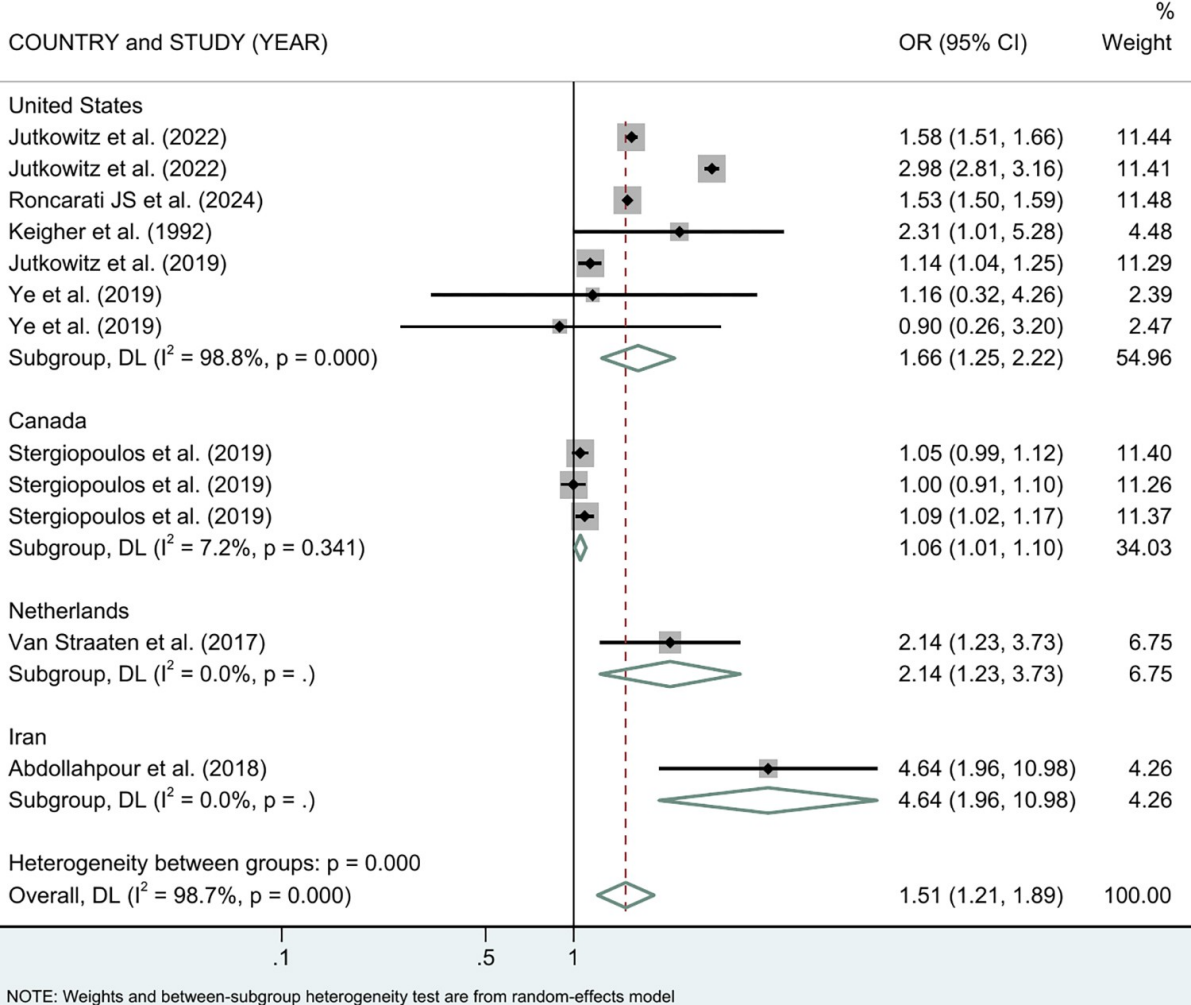
Meta-analysis of the association between PEH and NDDs stratified by countries.

We then analyzed the results by country subgroup and presented them in Fig 4. The United States had the largest number of studies included in the analysis. In the United States, PEH had a 66% higher risk of NDDs than non-PEH (OR = 1.66 (95% CI: 1.25, 2.22)). PEH in Canada also had a higher risk of NDDs, with an increase of 6% (OR = 1.06 (95% CI: 1.01, 1.10)). Such an increasing risk trend was also observed in other countries. In the Netherlands, the OR for PEH was higher than in the United States (OR = 2.14 (95% CI: 1.23, 3.73)), while Iran had the highest risk (OR = 4.64 (95% CI: 1.96, 10.98)).

## Discussion

This study shed new light by conducting the first meta-analysis to examine the association between PEH and multiple NDDs, as its concepts and themes. The main results are that among the most representative types of NDDs, PEH had a more than 50% increased risk of NDDs. As the current evidence available, some previous review articles have discussed this issue without calculating quantitative risk factors. For example, one review article identified PEH as a risk for ADRD by including nine articles [7], while another included 10 articles to assess the issue of cognitive impairment in PEH [25]. Some others have reviewed related studies from the opposite direction, noting that cognitive impairment is both a risk and a perpetuator of PEH [26]. Therefore, current research does not adequately address the relationship between PEH and NDDs or quantify their risks. The present study fills this research gap by its main finding of the significantly increased risk of NDDs among PEH (51%), with the specific risks for each NDD and each risk factor. The implication of this work can provide valuable insights for further research and policy-making to improve the health of PEH.

We combined our results and literature to explain the associations between NDD’s risk factors and PEH, which will contribute to the proposed implications. The reasons for the main finding of the 51% higher risks of NDDs in PEH may be complex and varied. One important possible explanation is the unfavorable living conditions that PEH often face. Compared to those with stable housing, PEH are often more exposed to adverse or extreme weather conditions, such as cold winters or hot summers [27, 28]. As a result, they may experience health problems such as frostbite, heat stroke, fever, injuries, skin diseases, inflamed wounds, and mental illness [27–31]. In Canada, it has been reported that the risk of hypothermia among PEH increases as temperatures drop [27]. Prolonged hypothermia can weaken the immune system, making people more susceptible to illnesses such as colds and fevers. Additionally, PEH face challenges in protecting themselves from infectious diseases [32]. As for the COVID-19 pandemic, which began in 2019 and lasted for several years, it spread widely despite government initiatives and calls to action, such as staying indoors or wearing masks [32, 33]. PEH confront challenges in adhering to self-protective measures such as maintaining social distance from others during epidemics, primarily due to their inhabitation in public environments [27]. Our previous research on the H1N1 virus in 2009 noted that PEH were potentially more vulnerable and susceptible to infectious disease transmission for these reasons [34, 35]. Such vulnerability and susceptibility of PEH to epidemics can lead to disease as well as sustained immune and inflammatory responses in the body and is one of the major factors in triggering NDDs [36].

Noise is an important contributor to the unfavorable living conditions of the PEH. Frequently chosen places for PEH include inside subways, tunnels, and around some public facilities [33]. While these locations serve to provide warmth and mitigate the risk of heat stroke during extreme weather conditions, they are frequently characterized by significant foot traffic. Noise from these foot traffic and commercial advertising is often sustained for longer periods and at higher decibels and can damage hearing. Hearing loss related to noise is associated with plastic changes in the brain and increased neural synchronization [37]. Prolonged noise exposure disrupts and even impairs PEH’s quality of sleep. Studies have shown that PEH experience poorer health due to sleep deprivation and poorer sleep quality [38]. Sleep deprivation and prolonged periods of light sleep may also compromise the PEH’s immune system. Noise increases levels of stress hormones and induces endothelial and neuronal dysfunction by mediating inflammatory and oxidative stress pathways. Chronic noise accompanied by oxidative stress disrupts neurogenesis in the hippocampus and impairs cognitive function in humans, which is a major cause of memory loss and AD [39]. This is consistent with our finding of a significantly increased risk of AD and ADRD in PEH.

Homelessness is a condition often associated with negative socioeconomic status (SES), such as unemployment, in addition to the negative effects of housing instability itself. Food insecurity and hunger can induce stress among PEH, with stress-related mental health issues such as anxiety exacerbating or perpetuating the problem [40]. As noted in the study published in NEJM, there is a relationship between food insecurity and health [40, 41]. Moreover, PEH frequently lacks the stability required for cooking, compounded by limited access to various ingredients due to SES. Consequently, PEH may experience undernourishment despite not being chronically hungry. Studies in the United States have found that PEH tend to have poor dietary habits, with a lack of fruit and vegetable intake, while mainly consuming sugar and fat [42]. Barriers to malnutrition have been found to include a lack of resources for cooking and storage, as well as an inability to afford more expensive and healthier foods such as fruits and vegetables [42]. Such nutritional deficiencies are also strongly associated with neurological dysfunction. For example, vitamin deficiencies have been implicated in several NDDs, including AD, Parkinson’s disease, Huntington’s chorea, and depression [43]. For example, thiamine (vitamin B1) deficiency has been associated with cognitive deficits, neurological deficits such as tau hyperphosphorylation, and AD-like pathology [44]. Mechanistically, AD is attributed to forming Aβ plaques due to vitamin B1, B12, and vitamin A deficiencies, whereas in MS, neuronal demyelination is attributed to vitamin C and vitamin D deficiencies [43]. This is consistent with our finding of a high risk of AD, dementia, and MS in the PEH. Thus, providing adequate nutrition to PEH may reduce the risk of multiple diseases, including NDDs, thereby reducing the potential social destabilization associated with neurological disorders as well as the significant societal burden of treating these mental illnesses.

Unfavorable SES limits PEH’s access to healthcare. According to a study in *The Lancet*, PEH had a higher prevalence of infectious diseases, mental disorders, and substance abuse [1]. Non-infectious diseases are prevalent among PEH, a factor thought to expedite the PEH’s aging process [1]. However, PEH with complex health conditions often lack access to primary healthcare [1]. PEH may lack access to medical care and knowledge of their illness stage. Our study’s findings on the high risk of NDDs among the PEH are crucial for societal decision-making. NDDs cause irreversible loss of neurons, and there are no effective treatments available. Therefore, early attention to the NDDs status of PEH is crucial for public health and reducing social burden.

A variety of significant psychosocial stressors often accompany PEH. PEH are vulnerable to chronic loneliness, unemployment-related stress, social exclusion, and exposure to violent environments, all of which are associated with mental health conditions such as anxiety and depression, particularly exacerbated by the COVID-19 pandemic [45, 46]. Mental health issues can induce stress in the nervous system, affect endocrinology, and even cause plaque buildup in blood vessels and nerves, potentially leading to the development of AD. For instance, chronic loneliness and social exclusion are significant contributors to depression, and prolonged depression can increase the risk of AD. Bioinformatics research suggests that loneliness and social isolation may lead to cognitive impairment and neurodegeneration. Studies validated that the gene related to loneliness significantly overlaps with the outcomes of AD and Parkinson’s disease (PD) in humans (82% and 68%, respectively) [47]. Additionally, anxiety has a strong physiological relationship with AD and impacts the early stages of the disease [48]. The high risk of AD and ADRD among PEH may be attributed to the chronic anxiety that often accompanies PEH, particularly in those with unfavorable SES and unemployment. These factors could manifest as a high risk of AD and ADRD, as indicated by our findings at the psychological level.

Our subgroup analyses identified PEH in various countries with different levels of risk for NDDs. The Netherlands, Iran, the United States, and Canada had the highest risk among the countries. PEH represent a higher prevalence for NDDs when PEH interact with aging. The experience of homelessness can induce psychological stress, impacting not only the onset of cognitive impairment but also potentially correlating with multiple sclerosis (MS) [49]. Individuals with MS have been found to have significantly higher loneliness scores, which may be attributed to their employment status. Other factors associated with MS include depression, cognitive and psychological fatigue, and lower psychological quality of life [49]. These support the high risk of MS associated with PEH in our findings.

PEH in Iran faced a higher risk of MS compared to those in other countries in this study. The risk of developing MS is significantly increased by negative lifestyle and conditions, including active and passive smoking, vitamin D deficiency, viral infections exposure, and immune system weakness [50]. These factors are often strongly associated with PEH and may contribute to the significant MS risk among PEH in Iran. Iran has experienced economic sanctions and political unrest for many years, leading to instability. Frequent conflicts and crises in the social environment have exacerbated poverty and unemployment, resulting in a rise in PEH. Moreover, these conflicts have laid bare societal infrastructure to environmental contamination and insecurity, potentially exacerbating the living conditions of PEH and increasing their vulnerability to infections and chronic illnesses. For instance, PEH who take drugs in Iran have a longer history and duration of injecting behavior [51]. Significant advancements in treating multiple sclerosis (MS) over the past two decades have enabled patients in high-income countries to effectively manage or even cure the disease through injections [52]. Studies demonstrate that immediate treatment post-diagnosis significantly enhances the prognosis of MS [53]. However, in low- and middle-income countries, accessing antibodies and therapeutic resources for MS is challenging, making the onset and progression of the disease difficult to manage. Financial constraints at the societal level make it difficult for PEH in Iran to receive timely medical support and treatment. This lack of timely access to MS treatment is one of the reasons why the risk of MS among PEH in Iran is significantly higher than in other countries [50, 53].

As two developed countries in North America that are often compared, we find that the risk of an increase in NDDs among PEH is higher in the United States than in Canada. The reasons for this difference may be multifactorial. First, comparative studies of the National Health Interview Survey (NHIS) in the United States and the Canadian Community Health Survey (CCHS) in Canada indicate that PEH in the United States with low SES have significantly worse health outcomes than their Canadian counterparts with similar conditions [54]. Canada presents better than the United States, possibly due to lower levels of health inequality and a population with better health and economic well-being [54]. In the United States, PEH with unfavorable SES encounter challenges in accessing healthcare services, which are typically commercialized. Their lack of public health insurance further exacerbates the difficulty in obtaining essential medical care and treatment. Only 26 percent of Americans are covered by public insurance, which is subjected to a considerably more complicated reimbursement process compared to the single-payer systems prevalent in Canada and Europe [55]. This is one of the significant barriers to maintaining the health of the PEH in the United States. The rapidly aging PEH have been found to have an increased prevalence of AD and ADRD in the United States [7]. Similar studies have focused on veteran populations and found that PEH are also a risk and consequence of ADRD [7]. The excess financial burden of PTSD alone has exceeded $232.2 billion for Americans, particularly United States veterans. On an individual level, the excess cost per person with PTSD is $19,630, along with the cost of treating other costly mental health disorders or long-term NDDs, which are generally unaffordable for the PEH [56]. On the other hand, although PEH are also an issue in Canada, Canada’s social assistance system and universal public health care system can provide free or low-cost basic health care to the PEH, making health care in Canada more accessible and equitable. Canada’s government-funded National Health Insurance (NHI) program provides universal health care to its citizens [55]. The social assistance system in Canada is more active than the United States, with the government offering housing assistance and other essential living supports for PEH. For instance, the Government of Canada spearheaded the development of the Homeless Individuals and Families Information System (HIFIS) to offer support and services for the day-to-day needs of PEH across the nation [57]. The Government of Canada not only focuses on collecting real-time data on PEH but also provides assistance in the form of shelter, bathing facilities, and referrals to other services [57]. This social support system alleviates the burden on PEH to some extent and reduces the duration of homelessness or extreme living conditions for PEH in Canada. This could potentially contribute to the significantly lower risk of NDDs among PEH in Canada compared to the United States, according to our findings.

Environmental factors may also contribute to the significantly higher risk of NDDs among PEH in the United States than in Canada. The United States has a serious problem with violent deaths. Compared to other high-income countries, the United States has a homicide rate that is seven times higher, while gun homicides are 25 times higher [58]. Compared to the United States, Canada has better safety and environmental conditions characterized by lower rates of shootings and violence. PEH already have adverse living conditions and challenges securing safe environments, even during nighttime. Coupled with enduring chronic stress and the fear of encountering gunfire and violence, it predisposes PEH to chronic fatigue and anxiety. Given the biological relationship between anxiety and AD, our findings support the increased risk of AD and dementia observed among PEH in the United States.

Canada, with its high immigration rate, incorporates immigrants who constitute 25% of its population, which underscores the notable ethnic diversity within the country [59]. Canada’s inclusive and diverse social environment, developed over time, helps alleviate stress, mental health burdens, and discrimination faced by immigrants and PEH to some extent, thereby reducing neurological damage or dysfunction associated with prolonged high levels of stress. This could be another contributing factor to the lower risk of NDDs among PEH in Canada compared to the United States in our findings [48].

Overall, this study found that PEH have a more than 50% increased risk of NDDs, particularly AD, dementia, cognitive impairment, and MS. The innovation is that we are the first to investigate the increased risk of multiple NDDs in PEH using a meta-analytic approach. We also analyzed the potential reasons for the different elevated risks of NDDs in different countries by subgroup analysis. Our findings and analyses address various perspectives regarding the differences in the NDDs’ risk among PEH in different countries, including SES factors, aging, environment, and healthcare systems. Our study helps bridge the research gap and offers valuable results for future studies and the formulation of health policies on this topic.

This study has several limitations. First, the lack of raw data made it difficult to perform more in-depth stratified analyses to understand the physiological mechanisms and other statistical characteristics, such as sex and age, behind this significantly increased risk of NDDs. In addition, the data for some countries or NDDs are still small. We only hope to offer a more comprehensive and representative combined risk assessment for NDDs by including as much data as possible. The studies included were mainly cross-sectional, which could be a potential limitation. Lastly, although efforts were made to encompass a wide range of common NDD types, some types, such as Parkinson’s disease, could not be analyzed due to a lack of raw data. We were unable to determine the extent to which some potentially confounding factors, such as substance use and psychiatric diseases, influenced the specific impact of PEH suffering from NDDs. This will be the focus of our future research. We also recommend further research on the risk of additional NDD types in PEH to better improve health, reduce the burden on society, and provide a scientific basis for academia and policymakers.

## Conclusion

In conclusion, homelessness has become an increasingly important public health challenge worldwide. The risks of NDDs among PEH have not been sufficiently discussed. Through a meta-analysis and systematic review of this topic, our findings indicate that PEH are at an elevated risk of NDDs in the order of multiple sclerosis, Alzheimer’s disease, cognitive impairment, and dementia. Furthermore, we found that this risk varies across countries. PEH in Iran, the Netherlands, the United States, and Canada are more susceptible to NDDs. Our study synthesized and discussed the factors contributing to such differences across countries, particularly focusing on aspects such as living environment and healthcare systems. Additionally, we provide new data support and research perspectives that can inspire future studies. There is a need for researchers and governments to pay more attention to the risk of NDDs among PEH and to formulate effective policies and measures to improve their living conditions and prevent the occurrence of NDDs. The enhanced research and policy will not only reduce the economic burden on society, particularly in terms of healthcare costs, but also uphold social stability and safeguard basic human rights.

## Supporting information

S1 Table Search criteria. (DOCX)

S2 Table Quality scores of eligible literatures. (DOCX)

S3 Table PRISMA checklist. (DOCX)

S1 Fig Begg’s test. (TIF)

S2 Fig Sensitivity analysis. (TIF)

S1 Text Conflicts of interest statement. (DOCX)
