# Supplementary material for: Associations between people experiencing homelessness (PEH) and neurodegenerative disorders (NDDs): A systematic review and meta-analysis
Source: PLoS One. 2024 Oct 22;19(10):e0312117. doi: 10.1371/journal.pone.0312117 (PMC11495621; doi:10.1371/journal.pone.0312117)
Supplement: S1 Table — (DOCX) [file pone.0312117.s001.docx]

**S1 Table – Search criteria**

- 1. **PubMed**

| **Query** | **Search Details** | **Results** |
| --- | --- | --- |
| "homeless", dementia | "homeless"[All Fields] AND ("dementia"[MeSH Terms] OR "dementia"[All Fields] OR "dementias"[All Fields] OR "dementia s"[All Fields]) | 45 |
| "homeless", alzheimer's | "homeless"[All Fields] AND ("alzheimer disease"[MeSH Terms] OR ("alzheimer"[All Fields] AND "disease"[All Fields]) OR "alzheimer disease"[All Fields] OR "alzheimer s"[All Fields]) | 9 |
| "homeless", neurodegenerative disorders | "homeless"[All Fields] AND ("neurodegenerative diseases"[MeSH Terms] OR ("neurodegenerative"[All Fields] AND "diseases"[All Fields]) OR "neurodegenerative diseases"[All Fields] OR ("neurodegenerative"[All Fields] AND "disorders"[All Fields]) OR "neurodegenerative disorders"[All Fields]) | 7 |
| "homeless", parkinson's | "homeless"[All Fields] AND ("parkinson disease"[MeSH Terms] OR ("parkinson"[All Fields] AND "disease"[All Fields]) OR "parkinson disease"[All Fields] OR "parkinson s"[All Fields]) | 4 |
| "homeless", cognitive impairment | "homeless"[All Fields] AND ("cognitive dysfunction"[MeSH Terms] OR ("cognitive"[All Fields] AND "dysfunction"[All Fields]) OR "cognitive dysfunction"[All Fields] OR ("cognitive"[All Fields] AND "impairment"[All Fields]) OR "cognitive impairment"[All Fields]) | 84 |
| "homeless", memory loss | "homeless"[All Fields] AND ("memory disorders"[MeSH Terms] OR ("memory"[All Fields] AND "disorders"[All Fields]) OR "memory disorders"[All Fields] OR ("memory"[All Fields] AND "loss"[All Fields]) OR "memory loss"[All Fields]) | 21 |
| "homeless", Cognitive dysfunction | "homeless"[All Fields] AND ("cognitive dysfunction"[MeSH Terms] OR ("cognitive"[All Fields] AND "dysfunction"[All Fields]) OR "cognitive dysfunction"[All Fields]) | 29 |
| "homeless", Cognitive disorders | "homeless"[All Fields] AND ("cognitive dysfunction"[MeSH Terms] OR ("cognitive"[All Fields] AND "dysfunction"[All Fields]) OR "cognitive dysfunction"[All Fields] OR ("cognitive"[All Fields] AND "disorders"[All Fields]) OR "cognitive disorders"[All Fields]) | 162 |
| "homeless", Neglect | "homeless"[All Fields] AND ("neglect"[All Fields] OR "neglected"[All Fields] OR "neglectful"[All Fields] OR "neglecting"[All Fields] OR "neglects"[All Fields]) | 178 |
| "homeless", Agnosia | "homeless"[All Fields] AND ("agnosia"[MeSH Terms] OR "agnosia"[All Fields] OR "agnosias"[All Fields]) | 0 |
| "homeless", Agnosia - Schema: all | "homeless"[All Fields] AND "Agnosia"[All Fields] | 0 |
| "homeless", Apraxia | "homeless"[All Fields] AND ("apraxias"[MeSH Terms] OR "apraxias"[All Fields] OR "apraxia"[All Fields]) | 0 |
| "homeless", Apraxia - Schema: all | "homeless"[All Fields] AND "Apraxia"[All Fields] | 0 |
| "homeless", Acalculia | "homeless"[All Fields] AND ("dyscalculia"[MeSH Terms] OR "dyscalculia"[All Fields] OR "acalculia"[All Fields]) | 0 |
| "homeless", Acalculia - Schema: all | "homeless"[All Fields] AND "Acalculia"[All Fields] | 0 |
| "homeless", Agraphia | "homeless"[All Fields] AND ("agraphia"[MeSH Terms] OR "agraphia"[All Fields] OR "agraphias"[All Fields]) | 0 |
| "homeless", Agraphia - Schema: all | "homeless"[All Fields] AND "Agraphia"[All Fields] | 0 |
| "homeless", Alexia | "homeless"[All Fields] AND ("dyslexia"[MeSH Terms] OR "dyslexia"[All Fields] OR "alexia"[All Fields] OR "alexias"[All Fields]) | 2 |
| "homeless", Aphasia | "homeless"[All Fields] AND ("aphasia"[MeSH Terms] OR "aphasia"[All Fields] OR "aphasias"[All Fields] OR "aphasia s"[All Fields]) | 1 |
| "homeless", Amnesia | "homeless"[All Fields] AND ("amnesia"[MeSH Terms] OR "amnesia"[All Fields] OR "amnesias"[All Fields]) | 1 |
| "homeless", MCI | "homeless"[All Fields] AND "mci"[All Fields] | 1 |
| "homeless", mild cognitive impairment | "homeless"[All Fields] AND ("cognitive dysfunction"[MeSH Terms] OR ("cognitive"[All Fields] AND "dysfunction"[All Fields]) OR "cognitive dysfunction"[All Fields] OR ("mild"[All Fields] AND "cognitive"[All Fields] AND "impairment"[All Fields]) OR "mild cognitive impairment"[All Fields]) | 31 |
| "homeless", AD | "homeless"[All Fields] AND ("administration and dosage"[MeSH Subheading] OR ("administration"[All Fields] AND "dosage"[All Fields]) OR "administration and dosage"[All Fields] OR "ad"[All Fields]) | 219 |
| "homeless", PD | "homeless"[All Fields] AND ("pharmacology"[MeSH Subheading] OR "pharmacology"[All Fields] OR "pd"[All Fields]) | 446 |
| "homeless", PD-related disorders | "homeless"[All Fields] AND ("PD-related"[All Fields] AND ("disease"[MeSH Terms] OR "disease"[All Fields] OR "disorder"[All Fields] OR "disorders"[All Fields] OR "disorder s"[All Fields] OR "disordes"[All Fields])) | 0 |
| "homeless", PD-related disorders - Schema: all | "homeless"[All Fields] AND ("PD-related"[All Fields] AND "disorders"[All Fields]) | 0 |
| "homeless", Motor neurone diseases | "homeless"[All Fields] AND (("motor"[All Fields] OR "motor s"[All Fields] OR "motoric"[All Fields] OR "motorically"[All Fields] OR "motorics"[All Fields] OR "motoring"[All Fields] OR "motorisation"[All Fields] OR "motorised"[All Fields] OR "motorization"[All Fields] OR "motorized"[All Fields] OR "motors"[All Fields]) AND ("neuron s"[All Fields] OR "neuronal"[All Fields] OR "neuronally"[All Fields] OR "neuronals"[All Fields] OR "neurone s"[All Fields] OR "neurones"[All Fields] OR "neuronic"[All Fields] OR "neurons"[MeSH Terms] OR "neurons"[All Fields] OR "neuron"[All Fields] OR "neurone"[All Fields]) AND ("disease"[MeSH Terms] OR "disease"[All Fields] OR "diseases"[All Fields] OR "disease s"[All Fields] OR "diseased"[All Fields])) | 0 |
| "homeless", Motor neurone diseases - Schema: all | "homeless"[All Fields] AND ("Motor"[All Fields] AND "neurone"[All Fields] AND "diseases"[All Fields]) | 0 |
| "homeless", Spinal muscular atrophy | "homeless"[All Fields] AND ("muscular atrophy, spinal"[MeSH Terms] OR ("muscular"[All Fields] AND "atrophy"[All Fields] AND "spinal"[All Fields]) OR "spinal muscular atrophy"[All Fields] OR ("spinal"[All Fields] AND "muscular"[All Fields] AND "atrophy"[All Fields])) | 0 |
| "homeless", Spinal muscular atrophy - Schema: all | "homeless"[All Fields] AND ("Spinal"[All Fields] AND "muscular"[All Fields] AND "atrophy"[All Fields]) | 0 |
| "homeless", prion diseases | "homeless"[All Fields] AND ("prion diseases"[MeSH Terms] OR ("prion"[All Fields] AND "diseases"[All Fields]) OR "prion diseases"[All Fields]) | 1 |
| "homeless", tauopathies | "homeless"[All Fields] AND ("tauopathies"[MeSH Terms] OR "tauopathies"[All Fields] OR "tauopathy"[All Fields]) | 2 |
| "homeless", multiple system atrophy | "homeless"[All Fields] AND ("multiple system atrophy"[MeSH Terms] OR ("multiple"[All Fields] AND "system"[All Fields] AND "atrophy"[All Fields]) OR "multiple system atrophy"[All Fields]) | 0 |
| "homeless", multiple system atrophy - Schema: all | "homeless"[All Fields] AND ("multiple"[All Fields] AND "system"[All Fields] AND "atrophy"[All Fields]) | 0 |
| "homeless", multiple sclerosis | "homeless"[All Fields] AND ("multiple sclerosis"[MeSH Terms] OR ("multiple"[All Fields] AND "sclerosis"[All Fields]) OR "multiple sclerosis"[All Fields]) | 2 |
| "homeless", amyotrophic lateral sclerosis | "homeless"[All Fields] AND ("amyotrophic lateral sclerosis"[MeSH Terms] OR ("amyotrophic"[All Fields] AND "lateral"[All Fields] AND "sclerosis"[All Fields]) OR "amyotrophic lateral sclerosis"[All Fields]) | 0 |
| "homeless", amyotrophic lateral sclerosis - Schema: all | "homeless"[All Fields] AND ("amyotrophic"[All Fields] AND "lateral"[All Fields] AND "sclerosis"[All Fields]) | 0 |
| "homeless", Progressive supranuclear palsy. | "homeless"[All Fields] AND ("supranuclear palsy, progressive"[MeSH Terms] OR ("supranuclear"[All Fields] AND "palsy"[All Fields] AND "progressive"[All Fields]) OR "progressive supranuclear palsy"[All Fields] OR ("progressive"[All Fields] AND "supranuclear"[All Fields] AND "palsy"[All Fields])) | 0 |
| "homeless", Progressive supranuclear palsy. - Schema: all | "homeless"[All Fields] AND ("Progressive"[All Fields] AND "supranuclear"[All Fields] AND "palsy"[All Fields]) | 0 |
| "homeless", Multiple system atrophy. | "homeless"[All Fields] AND ("multiple system atrophy"[MeSH Terms] OR ("multiple"[All Fields] AND "system"[All Fields] AND "atrophy"[All Fields]) OR "multiple system atrophy"[All Fields]) | 0 |
| "homeless", Multiple system atrophy. - Schema: all | "homeless"[All Fields] AND ("Multiple"[All Fields] AND "system"[All Fields] AND "atrophy"[All Fields]) | 0 |
| "homeless", Motor neuron disease. | "homeless"[All Fields] AND ("motor neuron disease"[MeSH Terms] OR ("motor"[All Fields] AND "neuron"[All Fields] AND "disease"[All Fields]) OR "motor neuron disease"[All Fields]) | 0 |
| "homeless", Motor neuron disease. - Schema: all | "homeless"[All Fields] AND ("Motor"[All Fields] AND "neuron"[All Fields] AND "disease"[All Fields]) | 0 |
| "homeless", Huntington's disease. | "homeless"[All Fields] AND ("huntington disease"[MeSH Terms] OR ("huntington"[All Fields] AND "disease"[All Fields]) OR "huntington disease"[All Fields] OR ("huntington s"[All Fields] AND "disease"[All Fields]) OR "huntington s disease"[All Fields]) | 1 |
| "homeless", Ataxia | "homeless"[All Fields] AND ("ataxia"[MeSH Terms] OR "ataxia"[All Fields] OR "ataxias"[All Fields]) | 1 |
| "homeless", memory disorders | "homeless"[All Fields] AND ("memory disorders"[MeSH Terms] OR ("memory"[All Fields] AND "disorders"[All Fields]) OR "memory disorders"[All Fields]) | 21 |
|  | Total | 1268 |

- 1. **Web of Science**

| **Entitlements** | **Type** | **Search Query** | **Database** | **Results** |
| --- | --- | --- | --- | --- |
| - WOS.IC: 1993 to 2024 - WOS.CCR: 1985 to 2024 - WOS.SCI: 1900 to 2024 - WOS.AHCI: 1975 to 2024 - WOS.BHCI: 2005 to 2024 - WOS.BSCI: 2005 to 2024 - WOS.ESCI: 2019 to 2024 - WOS.ISTP: 1990 to 2024 - WOS.SSCI: 1900 to 2024 - WOS.ISSHP: 1990 to 2024 | Search | homeless, dementia (All Fields) | Web of Science Core Collection | 50 |
| - WOS.IC: 1993 to 2024 - WOS.CCR: 1985 to 2024 - WOS.SCI: 1900 to 2024 - WOS.AHCI: 1975 to 2024 - WOS.BHCI: 2005 to 2024 - WOS.BSCI: 2005 to 2024 - WOS.ESCI: 2019 to 2024 - WOS.ISTP: 1990 to 2024 - WOS.SSCI: 1900 to 2024 - WOS.ISSHP: 1990 to 2024 | Search | homeless, alzheimer's (All Fields) | Web of Science Core Collection | 11 |
| - WOS.IC: 1993 to 2024 - WOS.CCR: 1985 to 2024 - WOS.SCI: 1900 to 2024 - WOS.AHCI: 1975 to 2024 - WOS.BHCI: 2005 to 2024 - WOS.BSCI: 2005 to 2024 - WOS.ESCI: 2019 to 2024 - WOS.ISTP: 1990 to 2024 - WOS.SSCI: 1900 to 2024 - WOS.ISSHP: 1990 to 2024 | Search | homeless, neurodegenerative disorders (All Fields) | Web of Science Core Collection | 2 |
| - WOS.IC: 1993 to 2024 - WOS.CCR: 1985 to 2024 - WOS.SCI: 1900 to 2024 - WOS.AHCI: 1975 to 2024 - WOS.BHCI: 2005 to 2024 - WOS.BSCI: 2005 to 2024 - WOS.ESCI: 2019 to 2024 - WOS.ISTP: 1990 to 2024 - WOS.SSCI: 1900 to 2024 - WOS.ISSHP: 1990 to 2024 | Search | homeless, parkinson's (All Fields) | Web of Science Core Collection | 2 |
| - WOS.IC: 1993 to 2024 - WOS.CCR: 1985 to 2024 - WOS.SCI: 1900 to 2024 - WOS.AHCI: 1975 to 2024 - WOS.BHCI: 2005 to 2024 - WOS.BSCI: 2005 to 2024 - WOS.ESCI: 2019 to 2024 - WOS.ISTP: 1990 to 2024 - WOS.SSCI: 1900 to 2024 - WOS.ISSHP: 1990 to 2024 | Search | homeless, cognitive impairment (All Fields) | Web of Science Core Collection | 107 |
| - WOS.IC: 1993 to 2024 - WOS.CCR: 1985 to 2024 - WOS.SCI: 1900 to 2024 - WOS.AHCI: 1975 to 2024 - WOS.BHCI: 2005 to 2024 - WOS.BSCI: 2005 to 2024 - WOS.ESCI: 2019 to 2024 - WOS.ISTP: 1990 to 2024 - WOS.SSCI: 1900 to 2024 - WOS.ISSHP: 1990 to 2024 | Search | homeless, memory loss (All Fields) | Web of Science Core Collection | 9 |
| - WOS.IC: 1993 to 2024 - WOS.CCR: 1985 to 2024 - WOS.SCI: 1900 to 2024 - WOS.AHCI: 1975 to 2024 - WOS.BHCI: 2005 to 2024 - WOS.BSCI: 2005 to 2024 - WOS.ESCI: 2019 to 2024 - WOS.ISTP: 1990 to 2024 - WOS.SSCI: 1900 to 2024 - WOS.ISSHP: 1990 to 2024 | Search | homeless, Cognitive dysfunction (All Fields) | Web of Science Core Collection | 24 |
| - WOS.IC: 1993 to 2024 - WOS.CCR: 1985 to 2024 - WOS.SCI: 1900 to 2024 - WOS.AHCI: 1975 to 2024 - WOS.BHCI: 2005 to 2024 - WOS.BSCI: 2005 to 2024 - WOS.ESCI: 2019 to 2024 - WOS.ISTP: 1990 to 2024 - WOS.SSCI: 1900 to 2024 - WOS.ISSHP: 1990 to 2024 | Search | homeless, Cognitive dysfunction (All Fields) | Web of Science Core Collection | 156 |
| - WOS.IC: 1993 to 2024 - WOS.CCR: 1985 to 2024 - WOS.SCI: 1900 to 2024 - WOS.AHCI: 1975 to 2024 - WOS.BHCI: 2005 to 2024 - WOS.BSCI: 2005 to 2024 - WOS.ESCI: 2019 to 2024 - WOS.ISTP: 1990 to 2024 - WOS.SSCI: 1900 to 2024 - WOS.ISSHP: 1990 to 2024 | Search | homeless, Neglect (All Fields) | Web of Science Core Collection | 318 |
| - WOS.IC: 1993 to 2024 - WOS.CCR: 1985 to 2024 - WOS.SCI: 1900 to 2024 - WOS.AHCI: 1975 to 2024 - WOS.BHCI: 2005 to 2024 - WOS.BSCI: 2005 to 2024 - WOS.ESCI: 2019 to 2024 - WOS.ISTP: 1990 to 2024 - WOS.SSCI: 1900 to 2024 - WOS.ISSHP: 1990 to 2024 | Search | homeless, Agnosia (All Fields) | Web of Science Core Collection | 0 |
| - WOS.IC: 1993 to 2024 - WOS.CCR: 1985 to 2024 - WOS.SCI: 1900 to 2024 - WOS.AHCI: 1975 to 2024 - WOS.BHCI: 2005 to 2024 - WOS.BSCI: 2005 to 2024 - WOS.ESCI: 2019 to 2024 - WOS.ISTP: 1990 to 2024 - WOS.SSCI: 1900 to 2024 - WOS.ISSHP: 1990 to 2024 | Search | homeless, Apraxia (All Fields) | Web of Science Core Collection | 0 |
| - WOS.IC: 1993 to 2024 - WOS.CCR: 1985 to 2024 - WOS.SCI: 1900 to 2024 - WOS.AHCI: 1975 to 2024 - WOS.BHCI: 2005 to 2024 - WOS.BSCI: 2005 to 2024 - WOS.ESCI: 2019 to 2024 - WOS.ISTP: 1990 to 2024 - WOS.SSCI: 1900 to 2024 - WOS.ISSHP: 1990 to 2024 | Search | homeless,Acalculia (All Fields) | Web of Science Core Collection | 0 |
| - WOS.IC: 1993 to 2024 - WOS.CCR: 1985 to 2024 - WOS.SCI: 1900 to 2024 - WOS.AHCI: 1975 to 2024 - WOS.BHCI: 2005 to 2024 - WOS.BSCI: 2005 to 2024 - WOS.ESCI: 2019 to 2024 - WOS.ISTP: 1990 to 2024 - WOS.SSCI: 1900 to 2024 - WOS.ISSHP: 1990 to 2024 | Search | homeless, Agraphia (All Fields) | Web of Science Core Collection | 0 |
| - WOS.IC: 1993 to 2024 - WOS.CCR: 1985 to 2024 - WOS.SCI: 1900 to 2024 - WOS.AHCI: 1975 to 2024 - WOS.BHCI: 2005 to 2024 - WOS.BSCI: 2005 to 2024 - WOS.ESCI: 2019 to 2024 - WOS.ISTP: 1990 to 2024 - WOS.SSCI: 1900 to 2024 - WOS.ISSHP: 1990 to 2024 | Search | homeless, Alexia (All Fields) | Web of Science Core Collection | 17 |
| - WOS.IC: 1993 to 2024 - WOS.CCR: 1985 to 2024 - WOS.SCI: 1900 to 2024 - WOS.AHCI: 1975 to 2024 - WOS.BHCI: 2005 to 2024 - WOS.BSCI: 2005 to 2024 - WOS.ESCI: 2019 to 2024 - WOS.ISTP: 1990 to 2024 - WOS.SSCI: 1900 to 2024 - WOS.ISSHP: 1990 to 2024 | Search | homeless, Aphasia (All Fields) | Web of Science Core Collection | 1 |
| - WOS.IC: 1993 to 2024 - WOS.CCR: 1985 to 2024 - WOS.SCI: 1900 to 2024 - WOS.AHCI: 1975 to 2024 - WOS.BHCI: 2005 to 2024 - WOS.BSCI: 2005 to 2024 - WOS.ESCI: 2019 to 2024 - WOS.ISTP: 1990 to 2024 - WOS.SSCI: 1900 to 2024 - WOS.ISSHP: 1990 to 2024 | Search | homeless, Amnesia (All Fields) | Web of Science Core Collection | 4 |
| - WOS.IC: 1993 to 2024 - WOS.CCR: 1985 to 2024 - WOS.SCI: 1900 to 2024 - WOS.AHCI: 1975 to 2024 - WOS.BHCI: 2005 to 2024 - WOS.BSCI: 2005 to 2024 - WOS.ESCI: 2019 to 2024 - WOS.ISTP: 1990 to 2024 - WOS.SSCI: 1900 to 2024 - WOS.ISSHP: 1990 to 2024 | Search | homeless, MCI (All Fields) | Web of Science Core Collection | 1 |
| - WOS.IC: 1993 to 2024 - WOS.CCR: 1985 to 2024 - WOS.SCI: 1900 to 2024 - WOS.AHCI: 1975 to 2024 - WOS.BHCI: 2005 to 2024 - WOS.BSCI: 2005 to 2024 - WOS.ESCI: 2019 to 2024 - WOS.ISTP: 1990 to 2024 - WOS.SSCI: 1900 to 2024 - WOS.ISSHP: 1990 to 2024 | Search | homeless, mild cognitive impairment (All Fields) | Web of Science Core Collection | 8 |
| - WOS.IC: 1993 to 2024 - WOS.CCR: 1985 to 2024 - WOS.SCI: 1900 to 2024 - WOS.AHCI: 1975 to 2024 - WOS.BHCI: 2005 to 2024 - WOS.BSCI: 2005 to 2024 - WOS.ESCI: 2019 to 2024 - WOS.ISTP: 1990 to 2024 - WOS.SSCI: 1900 to 2024 - WOS.ISSHP: 1990 to 2024 | Search | homeless, AD (All Fields) | Web of Science Core Collection | 143 |
| - WOS.IC: 1993 to 2024 - WOS.CCR: 1985 to 2024 - WOS.SCI: 1900 to 2024 - WOS.AHCI: 1975 to 2024 - WOS.BHCI: 2005 to 2024 - WOS.BSCI: 2005 to 2024 - WOS.ESCI: 2019 to 2024 - WOS.ISTP: 1990 to 2024 - WOS.SSCI: 1900 to 2024 - WOS.ISSHP: 1990 to 2024 | Search | homeless, PD (All Fields) | Web of Science Core Collection | 62 |
| - WOS.IC: 1993 to 2024 - WOS.CCR: 1985 to 2024 - WOS.SCI: 1900 to 2024 - WOS.AHCI: 1975 to 2024 - WOS.BHCI: 2005 to 2024 - WOS.BSCI: 2005 to 2024 - WOS.ESCI: 2019 to 2024 - WOS.ISTP: 1990 to 2024 - WOS.SSCI: 1900 to 2024 - WOS.ISSHP: 1990 to 2024 | Search | homeless, PD-related disorders (All Fields) | Web of Science Core Collection | 0 |
| - WOS.IC: 1993 to 2024 - WOS.CCR: 1985 to 2024 - WOS.SCI: 1900 to 2024 - WOS.AHCI: 1975 to 2024 - WOS.BHCI: 2005 to 2024 - WOS.BSCI: 2005 to 2024 - WOS.ESCI: 2019 to 2024 - WOS.ISTP: 1990 to 2024 - WOS.SSCI: 1900 to 2024 - WOS.ISSHP: 1990 to 2024 | Search | homeless, Motor neurone diseases (All Fields) | Web of Science Core Collection | 0 |
| - WOS.IC: 1993 to 2024 - WOS.CCR: 1985 to 2024 - WOS.SCI: 1900 to 2024 - WOS.AHCI: 1975 to 2024 - WOS.BHCI: 2005 to 2024 - WOS.BSCI: 2005 to 2024 - WOS.ESCI: 2019 to 2024 - WOS.ISTP: 1990 to 2024 - WOS.SSCI: 1900 to 2024 - WOS.ISSHP: 1990 to 2024 | Search | homeless, Spinal muscular atrophy (All Fields) | Web of Science Core Collection | 0 |
| - WOS.IC: 1993 to 2024 - WOS.CCR: 1985 to 2024 - WOS.SCI: 1900 to 2024 - WOS.AHCI: 1975 to 2024 - WOS.BHCI: 2005 to 2024 - WOS.BSCI: 2005 to 2024 - WOS.ESCI: 2019 to 2024 - WOS.ISTP: 1990 to 2024 - WOS.SSCI: 1900 to 2024 - WOS.ISSHP: 1990 to 2024 | Search | homeless, prion diseases (All Fields) | Web of Science Core Collection | 1 |
| - WOS.IC: 1993 to 2024 - WOS.CCR: 1985 to 2024 - WOS.SCI: 1900 to 2024 - WOS.AHCI: 1975 to 2024 - WOS.BHCI: 2005 to 2024 - WOS.BSCI: 2005 to 2024 - WOS.ESCI: 2019 to 2024 - WOS.ISTP: 1990 to 2024 - WOS.SSCI: 1900 to 2024 - WOS.ISSHP: 1990 to 2024 | Search | homeless, tauopathies (All Fields) | Web of Science Core Collection | 0 |
| - WOS.IC: 1993 to 2024 - WOS.CCR: 1985 to 2024 - WOS.SCI: 1900 to 2024 - WOS.AHCI: 1975 to 2024 - WOS.BHCI: 2005 to 2024 - WOS.BSCI: 2005 to 2024 - WOS.ESCI: 2019 to 2024 - WOS.ISTP: 1990 to 2024 - WOS.SSCI: 1900 to 2024 - WOS.ISSHP: 1990 to 2024 | Search | homeless, multiple system atrophy (All Fields) | Web of Science Core Collection | 0 |
| - WOS.IC: 1993 to 2024 - WOS.CCR: 1985 to 2024 - WOS.SCI: 1900 to 2024 - WOS.AHCI: 1975 to 2024 - WOS.BHCI: 2005 to 2024 - WOS.BSCI: 2005 to 2024 - WOS.ESCI: 2019 to 2024 - WOS.ISTP: 1990 to 2024 - WOS.SSCI: 1900 to 2024 - WOS.ISSHP: 1990 to 2024 | Search | homeless, multiple sclerosis (All Fields) | Web of Science Core Collection | 4 |
| - WOS.IC: 1993 to 2024 - WOS.CCR: 1985 to 2024 - WOS.SCI: 1900 to 2024 - WOS.AHCI: 1975 to 2024 - WOS.BHCI: 2005 to 2024 - WOS.BSCI: 2005 to 2024 - WOS.ESCI: 2019 to 2024 - WOS.ISTP: 1990 to 2024 - WOS.SSCI: 1900 to 2024 - WOS.ISSHP: 1990 to 2024 | Search | homeless, amyotrophic lateral sclerosis (All Fields) | Web of Science Core Collection | 0 |
| - WOS.IC: 1993 to 2024 - WOS.CCR: 1985 to 2024 - WOS.SCI: 1900 to 2024 - WOS.AHCI: 1975 to 2024 - WOS.BHCI: 2005 to 2024 - WOS.BSCI: 2005 to 2024 - WOS.ESCI: 2019 to 2024 - WOS.ISTP: 1990 to 2024 - WOS.SSCI: 1900 to 2024 - WOS.ISSHP: 1990 to 2024 | Search | homeless, Progressive supranuclear palsy. (All Fields) | Web of Science Core Collection | 0 |
| - WOS.IC: 1993 to 2024 - WOS.CCR: 1985 to 2024 - WOS.SCI: 1900 to 2024 - WOS.AHCI: 1975 to 2024 - WOS.BHCI: 2005 to 2024 - WOS.BSCI: 2005 to 2024 - WOS.ESCI: 2019 to 2024 - WOS.ISTP: 1990 to 2024 - WOS.SSCI: 1900 to 2024 - WOS.ISSHP: 1990 to 2024 | Search | homeless, Multiple system atrophy. (All Fields) | Web of Science Core Collection | 0 |
| - WOS.IC: 1993 to 2024 - WOS.CCR: 1985 to 2024 - WOS.SCI: 1900 to 2024 - WOS.AHCI: 1975 to 2024 - WOS.BHCI: 2005 to 2024 - WOS.BSCI: 2005 to 2024 - WOS.ESCI: 2019 to 2024 - WOS.ISTP: 1990 to 2024 - WOS.SSCI: 1900 to 2024 - WOS.ISSHP: 1990 to 2024 | Search | homeless, Motor neuron disease. (All Fields) | Web of Science Core Collection | 1 |
| - WOS.IC: 1993 to 2024 - WOS.CCR: 1985 to 2024 - WOS.SCI: 1900 to 2024 - WOS.AHCI: 1975 to 2024 - WOS.BHCI: 2005 to 2024 - WOS.BSCI: 2005 to 2024 - WOS.ESCI: 2019 to 2024 - WOS.ISTP: 1990 to 2024 - WOS.SSCI: 1900 to 2024 - WOS.ISSHP: 1990 to 2024 | Search | homeless, Huntington's disease. (All Fields) | Web of Science Core Collection | 0 |
| - WOS.IC: 1993 to 2024 - WOS.CCR: 1985 to 2024 - WOS.SCI: 1900 to 2024 - WOS.AHCI: 1975 to 2024 - WOS.BHCI: 2005 to 2024 - WOS.BSCI: 2005 to 2024 - WOS.ESCI: 2019 to 2024 - WOS.ISTP: 1990 to 2024 - WOS.SSCI: 1900 to 2024 - WOS.ISSHP: 1990 to 2024 | Search | homeless, Ataxia (All Fields) | Web of Science Core Collection | 1 |
| - WOS.IC: 1993 to 2024 - WOS.CCR: 1985 to 2024 - WOS.SCI: 1900 to 2024 - WOS.AHCI: 1975 to 2024 - WOS.BHCI: 2005 to 2024 - WOS.BSCI: 2005 to 2024 - WOS.ESCI: 2019 to 2024 - WOS.ISTP: 1990 to 2024 - WOS.SSCI: 1900 to 2024 - WOS.ISSHP: 1990 to 2024 | Search | homeless, memory disorders (All Fields) | Web of Science Core Collection | 37 |
|  |  |  | Total | 959 |

- 1. **PsycINFO**

| Searched for | Databases | Results |
| --- | --- | --- |
| "homeless", Amnesia | APA PsycInfo® | 6 |
| noft("homeless", alzheimer's) | APA PsycInfo® | 10 |
| noft("homeless", neurodegenerative disorders) | APA PsycInfo® | 0 |
| "homeless", parkinson's | APA PsycInfo® | 1 |
| noft("homeless", cognitive impairment) | APA PsycInfo® | 97 |
| noft("homeless", memory loss) | APA PsycInfo® | 14 |
| noft("homeless", Cognitive dysfunction) | APA PsycInfo® | 28 |
| noft("homeless", Agnosia) | APA PsycInfo® | 0 |
| "homeless", Agnosia - Schema: all | APA PsycInfo® | 0 |
| "homeless", Apraxia | APA PsycInfo® | 1 |
| noft("homeless", Apraxia - Schema: all) | APA PsycInfo® | 0 |
| "homeless", Acalculia | APA PsycInfo® | 0 |
| "homeless", Acalculia - Schema: all | APA PsycInfo® | 0 |
| "homeless", Agraphia | APA PsycInfo® | 0 |
| "homeless", Agraphia - Schema: all | APA PsycInfo® | 0 |
| "homeless", Alexia | APA PsycInfo® | 10 |
| noft("homeless", Aphasia) | APA PsycInfo® | 1 |
| noft("homeless", Amnesia) | APA PsycInfo® | 6 |
| noft("homeless", MCI) | APA PsycInfo® | 1 |
| noft("homeless", mild cognitive impairment) | APA PsycInfo® | 10 |
| noft("homeless", AD) | APA PsycInfo® | 16 |
| noft("homeless", PD) | APA PsycInfo® | 9 |
| noft("homeless", PD-related disorders) | APA PsycInfo® | 0 |
| "homeless", Motor neurone diseases | APA PsycInfo® | 0 |
| "homeless", Spinal muscular atrophy | APA PsycInfo® | 0 |
| "homeless", prion diseases | APA PsycInfo® | 1 |
| noft("homeless", tauopathies) | APA PsycInfo® | 0 |
| "homeless", multiple system atrophy | APA PsycInfo® | 0 |
| "homeless", multiple sclerosis | APA PsycInfo® | 2 |
| noft("homeless", Progressive supranuclear palsy.) | APA PsycInfo® | 0 |
| "homeless", Multiple system atrophy. | APA PsycInfo® | 0 |
| "homeless", Motor neuron disease. | APA PsycInfo® | 0 |
| "homeless", Huntington's disease. | APA PsycInfo® | 3 |
| noft("homeless", Ataxia) | APA PsycInfo® | 0 |
|  | Total | 216 |

- 1. **Scopus**

| serching terms | RESULTS |
| --- | --- |
| TITLE-ABS-KEY ( "homeless" , AND huntington's AND disease. ) | 2 |
| TITLE-ABS-KEY ( "homeless" , AND motor AND neuron AND disease. ) | 2 |
| TITLE-ABS-KEY ( "homeless" , AND multiple AND system AND atrophy. ) | 2 |
| TITLE-ABS-KEY ( "homeless" , AND multiple AND sclerosis ) | 0 |
| TITLE-ABS-KEY ( "homeless" , AND multiple AND system AND atrophy ) | 9 |
| TITLE-ABS-KEY ( "homeless" , AND prion AND diseases ) | 0 |
| TITLE-ABS-KEY ( "homeless" , AND spinal AND muscular AND atrophy ) | 0 |
| TITLE-ABS-KEY ( "homeless" , AND motor AND neurone AND diseases ) | 0 |
| TITLE-ABS-KEY ( "homeless" , AND pd-related AND disorders ) | 0 |
| TITLE-ABS-KEY ( "homeless" , AND pd ) | 8 |
| TITLE-ABS-KEY ( "homeless" , AND ad ) | 50 |
| TITLE-ABS-KEY ( "homeless" , AND mild AND cognitive AND impairment ) | 9 |
| TITLE-ABS-KEY ( "homeless" , AND mci ) | 2 |
| TITLE-ABS-KEY ( "homeless" , AND aphasia ) | 4 |
| TITLE-ABS-KEY ( "homeless" , AND alexia ) | 0 |
| TITLE-ABS-KEY ( "homeless" , AND agraphia ) | 0 |
| TITLE-ABS-KEY ( "homeless" , AND acalculia ) | 0 |
| TITLE-ABS-KEY ( "homeless" , AND apraxia ) | 2 |
| TITLE-ABS-KEY ( "homeless" , AND agnosia ) | 1 |
| TITLE-ABS-KEY ( "homeless" , AND cognitive AND dysfunction ) | 46 |
| TITLE-ABS-KEY ( "homeless" , AND memory AND loss ) | 20 |
| TITLE-ABS-KEY ( "homeless" , AND cognitive AND impairment ) | 103 |
| TITLE-ABS-KEY ( "homeless" , AND parkinson's ) | 10 |
| TITLE-ABS-KEY ( "homeless" , AND amnesia ) | 15 |
| TITLE-ABS-KEY ( homeless, AND dementia ) | 95 |
| TITLE-ABS-KEY ( homeless, AND dementia ) | 95 |
| ( TITLE-ABS-KEY ( "homeless" , AND amnesia ) ) AND ( TITLE-ABS-KEY ( homeless, AND dementia ) ) | 1 |
| TITLE-ABS-KEY ( "homeless" , AND amnesia ) | 15 |
| TITLE-ABS-KEY ( homeless, AND dementia ) | 95 |
| Total | 586 |
