# Supplementary material for: Associations between people experiencing homelessness (PEH) and neurodegenerative disorders (NDDs): A systematic review and meta-analysis
Source: PLoS One. 2024 Oct 22;19(10):e0312117. doi: 10.1371/journal.pone.0312117 (PMC11495621; doi:10.1371/journal.pone.0312117)
Supplement: S2 Table — (DOCX) [file pone.0312117.s002.docx]

**S2 Table – Quality scores of eligible literatures**

| **STUDY** | **YEAR** | **Indicated the age group** | **Indicated the design of study (eg, cohort study)** | **Indicated the source of data (eg, the Center for Medicaid and Medicare)** | **Discussed inclusion and exclusion information for data** | **Indicated the demographics of location (eg, Canada or United States)** | **Indicated the patients data information** | **Described any assessment undertaken for quality assurance purposes (eg, sensitivity analysis)** | **Listed criteria for excluding certain data during analysis** | **Described how confounding was assessed and/or controlled (eg, results were adjusted for age)** | **Explained whether missing data were handled in the analysis** | **Total Score** |
| --- | --- | --- | --- | --- | --- | --- | --- | --- | --- | --- | --- | --- |
| Jutkowitz et al. | 2021 | 1 | 1 | 1 | 1 | 1 | 1 | 0 | 0 | 1 | 0 | 7 |
| Roncarati JS et al. | 2024 | 1 | 1 | 1 | 1 | 1 | 1 | 1 | 1 | 1 | 0 | 9 |
| Keigher et al. | 1992 | 1 | 1 | 1 | 1 | 1 | 1 | 0 | 0 | 0 | 0 | 6 |
| Jutkowitz et al. | 2019 | 1 | 1 | 1 | 1 | 1 | 1 | 0 | 1 | 1 | 0 | 8 |
| Ye et al. | 2019 | 1 | 1 | 1 | 1 | 1 | 1 | 0 | 1 | 0 | 0 | 7 |
| Stergiopoulos et al. | 2019 | 1 | 0 | 1 | 1 | 1 | 1 | 0 | 1 | 1 | 0 | 7 |
| Straaten et al. | 2017 | 1 | 1 | 1 | 1 | 1 | 1 | 0 | 1 | 1 | 1 | 9 |
| Abdollahpour et al. | 2018 | 1 | 1 | 1 | 1 | 1 | 1 | 0 | 0 | 1 | 0 | 7 |
