# Supplementary figures and images for: Associations between people experiencing homelessness (PEH) and neurodegenerative disorders (NDDs): A systematic review and meta-analysis
Source: PLoS One. 2024 Oct 22;19(10):e0312117. doi: 10.1371/journal.pone.0312117 (PMC11495621; doi:10.1371/journal.pone.0312117)

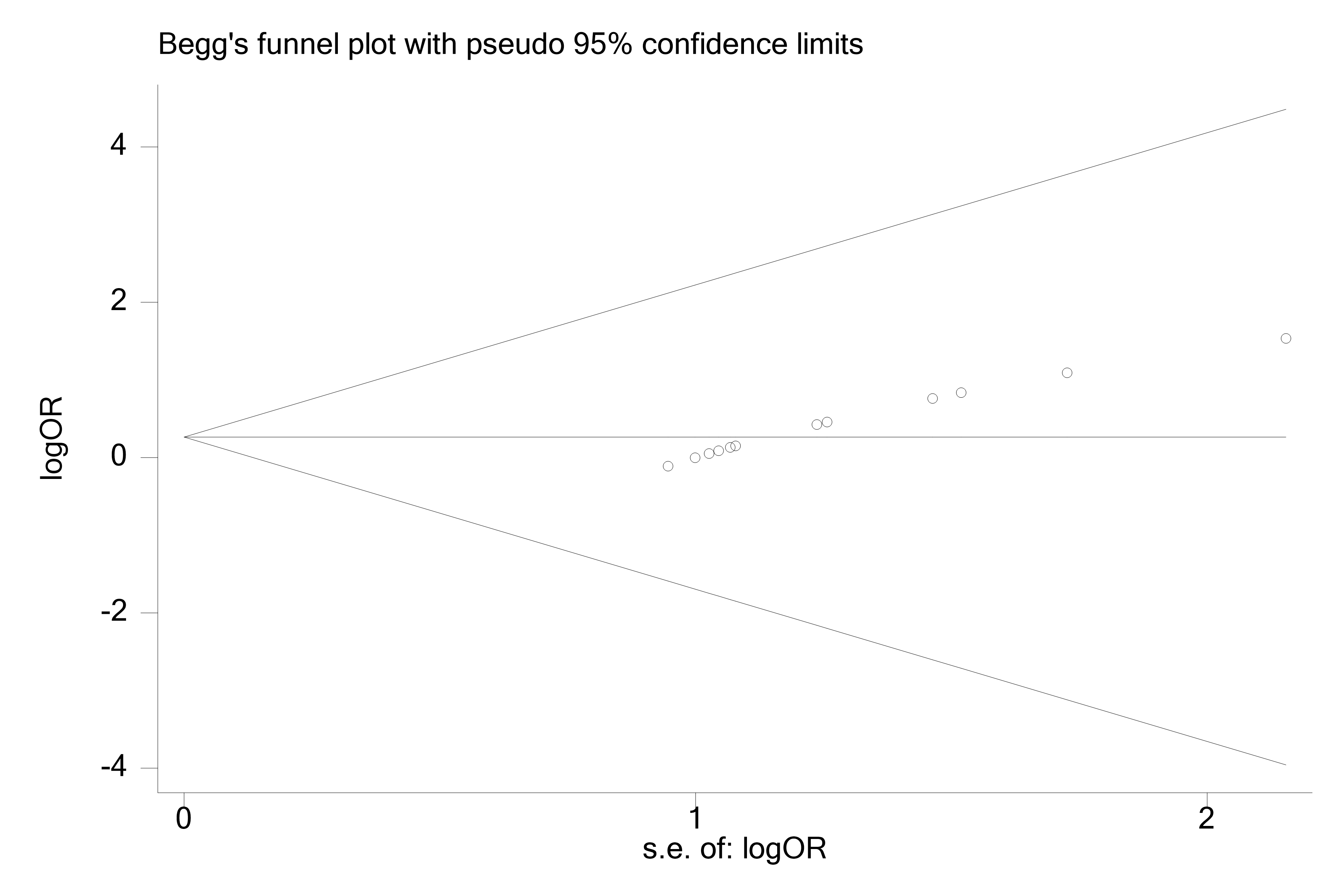

Supplement: S1 Fig — (TIF) [file pone.0312117.s004.tif]

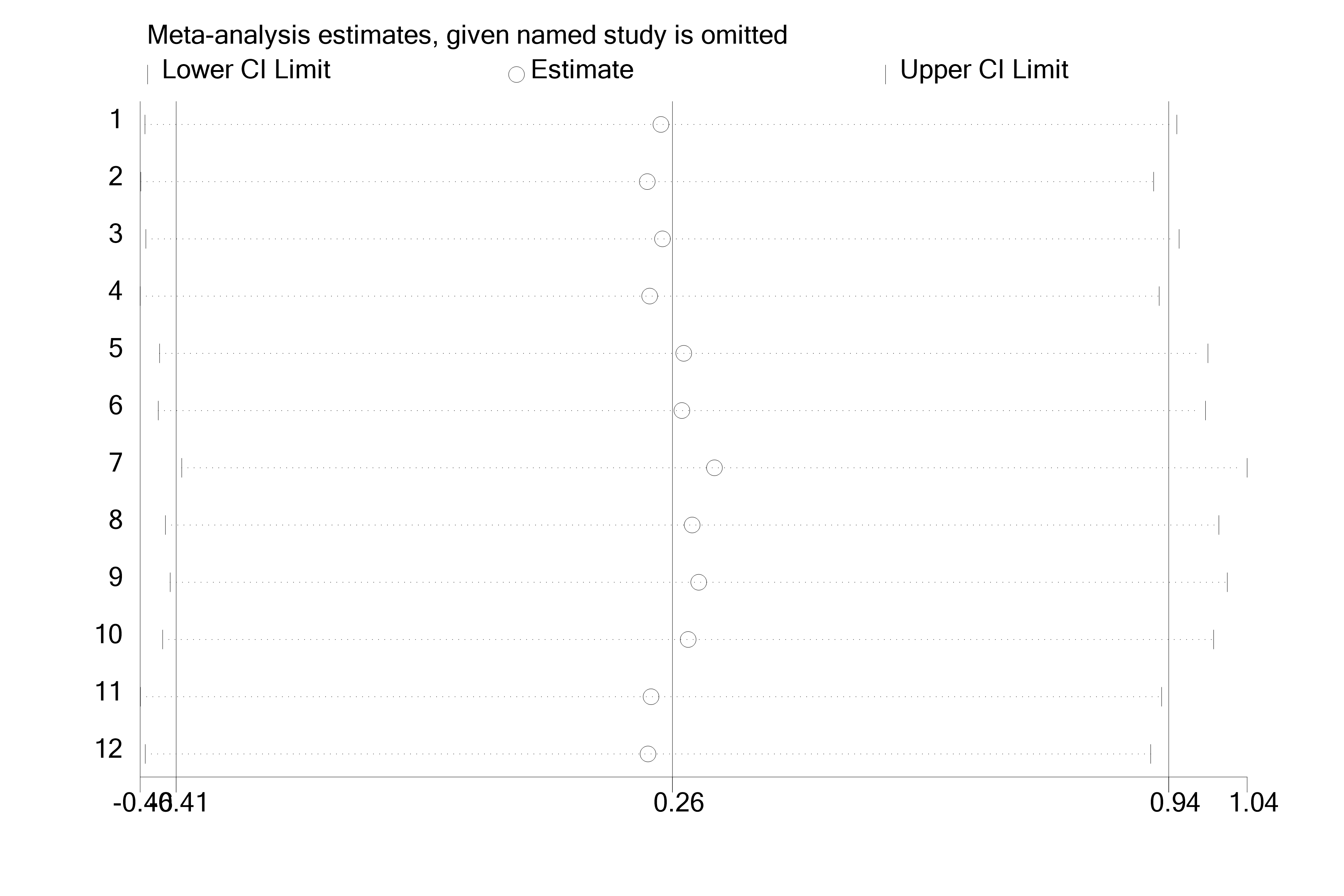

Supplement: S2 Fig — (TIF) [file pone.0312117.s005.tif]
