# Supplementary material for: Associations between people experiencing homelessness (PEH) and neurodegenerative disorders (NDDs): A systematic review and meta-analysis
Source: PLoS One. 2024 Oct 22;19(10):e0312117. doi: 10.1371/journal.pone.0312117 (PMC11495621; doi:10.1371/journal.pone.0312117)
Supplement: S1 Text — (DOCX) [file pone.0312117.s006.docx]

**S1 Text – Conflicts of interest statement**

The authors declare that there are no competing or conflicts of interest. There is no registration for the review, protocol, or any amendments to the information provided.

**Table - Excluded studies**

| Title | DOI | Excluded Reasons |
| --- | --- | --- |
| Outcomes of psychosocial interventions for homeless individuals with mental illness: A systematic review | [DOI: 10.1177/00207640231217173](https://pubmed.ncbi.nlm.nih.gov/38174711/) | It is review paper |
| Criminal behavior and victimization among homeless individuals with severe mental illness: a systematic review | [DOI: 10.1176/appi.ps.201200515](https://pubmed.ncbi.nlm.nih.gov/24535245/) | It is review paper |
| Commentary on 'Outcomes of psychosocial interventions for homeless individuals with mental illness: A systematic review' by Roniyamol Roy et al | DOI: 10.1177/00207640241250308 | It is review paper |
| The prevalence of mental illness in homeless children: a systematic review and meta-analysis | DOI: 10.1016/j.jaac.2014.11.008 | It is review paper |
| The Impact of Housing First on Criminal Justice Outcomes among Homeless People with Mental Illness: A Systematic Review | DOI: 10.1177/0706743718815902 | It is review paper |
| The physical and mental health effects of housing homeless people: A systematic review | DOI: 10.1111/hsc.13486 | It is review paper |
| Trajectories and mental health-related predictors of perceived discrimination and stigma among homeless adults with mental illness | DOI: 10.1371/journal.pone.0229385 | Abstract did not contain calculable risk estimates such as ORs (Odds Ratios) |
| Mental Health Conservatorship Among Homeless People With Serious Mental Illness | DOI: 10.1176/appi.ps.202100254 | Abstract did not contain calculable risk estimates such as ORs (Odds Ratios) |
| Mental illness among 500 people living homeless and referred for psychiatric evaluation in Lisbon, Portugal | DOI: 10.1017/S1092852921000547 | Abstract did not contain calculable risk estimates such as ORs (Odds Ratios) |
| Homelessness and Mental Illness | DOI: 10.1097/PRA.0000000000000688 | Abstract did not contain calculable risk estimates such as ORs (Odds Ratios) |
| Caring for homeless persons with serious mental illness in general hospitals | DOI: 10.1016/j.psym.2012.10.004 | Abstract did not contain calculable risk estimates such as ORs (Odds Ratios) |
| Homeless persons with mental illness and COVID pandemic: Collective efforts from India | DOI: 10.1016/j.ajp.2020.102268 | Abstract did not contain calculable risk estimates such as ORs (Odds Ratios) |
| Chronic Pain Among Homeless Persons with Mental Illness | DOI: 10.1093/pm/pnw324 | Abstract did not contain calculable risk estimates such as ORs (Odds Ratios) |
| Predictors of Mental Health Recovery in Homeless Adults with Mental Illness | DOI: 10.1007/s10597-018-0356-3 | Abstract did not contain calculable risk estimates such as ORs (Odds Ratios) |
| Prevalence of Mental Illness among Homeless People in Hong Kong | DOI: 10.1371/journal.pone.0140940 | Abstract did not contain calculable risk estimates such as ORs (Odds Ratios) |
| Perspectives of community members on homeless people with mental illness in Nsawam, Ghana | DOI: 10.1177/0020764020984195 | Abstract did not contain calculable risk estimates such as ORs (Odds Ratios) |
| Characteristics of Individuals With Mental Illness in Tokyo Homeless Shelters. | DOI: 10.1176/appi.ps.201400517 | Abstract did not contain calculable risk estimates such as ORs (Odds Ratios) |
| Sexual Intimacy, Mental Illness, and Homelessness | DOI: 10.1002/ajcp.12213 | Abstract did not contain calculable risk estimates such as ORs (Odds Ratios) |
| Factors associated with higher healthcare costs in a cohort of homeless adults with a mental illness and a general cohort of adults with a history of homelessness | DOI: 10.1186/s12913-021-06562-6 | Abstract did not contain calculable risk estimates such as ORs (Odds Ratios) |
| Homeless Patients Associate Clinician Bias With Suboptimal Care for Mental Illness, Addictions, and Chronic Pain | DOI: 10.1177/2150132720910289 | Abstract did not contain calculable risk estimates such as ORs (Odds Ratios) |
| Beyond Supported Housing: Correlates of Improvements in Quality of Life Among Homeless Adults with Mental Illness | DOI: 10.1007/s11126-022-10010-x | Abstract did not contain calculable risk estimates such as ORs (Odds Ratios) |
| Coping amidst an assemblage of disadvantage: A qualitative metasynthesis of first-person accounts of managing severe mental illness while homeless | DOI: 10.1111/jpm.12524 | Abstract did not contain calculable risk estimates such as ORs (Odds Ratios) |
| Service use and recovery among currently and formerly homeless adults with mental illness | DOI: 10.1177/0020764020913324 | Abstract did not contain calculable risk estimates such as ORs (Odds Ratios) |
| Stakeholders facilitating hope and empowerment amidst social suffering: A qualitative documentary analysis exploring lives of homeless women with mental illness | DOI: 10.1177/00207640211011186 | Abstract did not contain calculable risk estimates such as ORs (Odds Ratios) |
| Eventful past, stagnant present, and hopeful future: A time order analysis of experiences of homeless women with chronic mental illness residing in shelter care homes | DOI: 10.1177/00207640211060148 | Abstract did not contain calculable risk estimates such as ORs (Odds Ratios) |
| Problem solving skills and deficits among homeless veterans with serious mental illness | DOI: 10.1037/ort0000340 | Abstract did not contain calculable risk estimates such as ORs (Odds Ratios) |
| Mental illness and housing outcomes among a sample of homeless men in an Australian urban centre | DOI: 10.1177/0004867414563187 | Abstract did not contain calculable risk estimates such as ORs (Odds Ratios) |
| Problems of epidemiologic method in assessing the type and extent of mental illness among homeless adults | DOI: 10.1176/ps.40.3.261 | Abstract did not contain calculable risk estimates such as ORs (Odds Ratios) |
| Mental illness among homeless individuals in a suburban county | DOI: 10.1176/ps.48.4.504 | Abstract did not contain calculable risk estimates such as ORs (Odds Ratios) |
| Identifying Social Skills That Support Housing Attainment and Retention Among Homeless Persons With Serious Mental Illness | DOI: 10.1176/appi.ps.201800508 | Abstract did not contain calculable risk estimates such as ORs (Odds Ratios) |
| Effectiveness of Housing First for Homeless Adults with Mental Illness Who Frequently Use Emergency Departments in a Multisite Randomized Controlled Trial | DOI: 10.1007/s10488-020-01008-3 | Abstract did not contain calculable risk estimates such as ORs (Odds Ratios) |
| Homelessness in schizophrenia | DOI: 10.1016/j.psc.2012.06.010 | Abstract did not contain calculable risk estimates such as ORs (Odds Ratios) |
| Financial Strain, Mental Illness, and Homelessness: Results From a National Longitudinal Study | DOI: 10.1097/MLR.0000000000001453 | Abstract did not contain calculable risk estimates such as ORs (Odds Ratios) |
| Prevalence of mental illness, intellectual disability, and developmental disability among homeless people in Nagoya, Japan: A case series study | DOI: 10.1111/pcn.12265 | Abstract did not contain calculable risk estimates such as ORs (Odds Ratios) |
| Homelessness and mental illness | DOI: 10.1192/bjp.162.3.314 | Abstract did not contain calculable risk estimates such as ORs (Odds Ratios) |
| Continuity of primary care among homeless adults with mental illness who received a housing and mental health intervention | DOI: 10.1093/fampra/cmad023 | Abstract did not contain calculable risk estimates such as ORs (Odds Ratios) |
| Effect of a Housing Intervention on Selected Cardiovascular Risk Factors Among Homeless Adults With Mental Illness: 24-Month Follow-Up of a Randomized Controlled Trial | DOI: 10.1161/JAHA.119.016896 | Abstract did not contain calculable risk estimates such as ORs (Odds Ratios) |
| The Ties That Bind and Unbound Ties: Experiences of Formerly Homeless Individuals in Recovery From Serious Mental Illness and Substance Use | DOI: 10.1177/1049732318814250 | Abstract did not contain calculable risk estimates such as ORs (Odds Ratios) |
| Persisting Barriers to Employment for Recently Housed Adults with Mental Illness Who Were Homeless | DOI: 10.1007/s11524-015-0012-y | Abstract did not contain calculable risk estimates such as ORs (Odds Ratios) |
| Comparison of homeless clinic attenders with and without psychotic illness | DOI: 10.1177/0004867419893426 | Abstract did not contain calculable risk estimates such as ORs (Odds Ratios) |
| Mental illness in homeless women: an epidemiological study in Munich, Germany | DOI: 10.1007/BF03033070 | Abstract did not contain calculable risk estimates such as ORs (Odds Ratios) |
| Pathways to social integration among homeless-experienced adults with serious mental illness: a qualitative perspective | DOI: 10.1186/s12913-024-11678-6 | Abstract did not contain calculable risk estimates such as ORs (Odds Ratios) |
| Trajectories of Recovery Among Formerly Homeless Adults With Serious Mental Illness | DOI: 10.1176/appi.ps.201500126 | Abstract did not contain calculable risk estimates such as ORs (Odds Ratios) |
| History of foster care among homeless adults with mental illness in Vancouver, British Columbia: a precursor to trajectories of risk | DOI: 10.1186/s12888-015-0411-3 | Abstract did not contain calculable risk estimates such as ORs (Odds Ratios) |
| Policy Recommendations to Address Housing Shortages for People With Severe Mental Illness | DOI: 10.1176/appi.ps.202100158 | Abstract did not contain calculable risk estimates such as ORs (Odds Ratios) |
| Mental illness and substance use problems in relation to homelessness onset | DOI: 10.5993/AJHB.39.4.11 | Abstract did not contain calculable risk estimates such as ORs (Odds Ratios) |
| Quality of life of homeless persons with mental illness: results from the course-of-homelessness study | DOI: 10.1176/appi.ps.51.9.1135 | Abstract did not contain calculable risk estimates such as ORs (Odds Ratios) |
| Psychopharmacologic Services for Homeless Veterans: Comparing Psychotropic Prescription Fills Among Homeless and Non-Homeless Veterans with Serious Mental Illness | DOI: 10.1007/s10597-015-9904-2 | Abstract did not contain calculable risk estimates such as ORs (Odds Ratios) |
| Risk of mental ill-health among homeless women in Madrid (Spain) | DOI: 10.1007/s00737-020-01036-w | Abstract did not contain calculable risk estimates such as ORs (Odds Ratios) |
| Placement of chronically homeless into different types of permanent supportive housing before and after a coordinated entry system: The influence of severe mental illness, substance use disorder, and dual diagnosis on housing configuration and intensity of services | DOI: 10.1002/jcop.22428 | Abstract did not contain calculable risk estimates such as ORs (Odds Ratios) |
| Health and social adjustment of homeless older adults with a mental illness | DOI: 10.1176/appi.ps.201100175 | Abstract did not contain calculable risk estimates such as ORs (Odds Ratios) |
| Predictors of criminal justice system trajectories of homeless adults living with mental illness | DOI: 10.1016/j.ijlp.2016.05.013 | Abstract did not contain calculable risk estimates such as ORs (Odds Ratios) |
| Insights from homeless men about PRISM, an innovative shelter-based mental health service | DOI: 10.1371/journal.pone.0250341 | Abstract did not contain calculable risk estimates such as ORs (Odds Ratios) |
| A history in-care predicts unique characteristics in a homeless population with mental illness | DOI: 10.1016/j.chiabu.2013.08.018 | Abstract did not contain calculable risk estimates such as ORs (Odds Ratios) |
| Social support and housing transitions among homeless adults with serious mental illness and substance use disorders | DOI: 10.1037/prj0000213 | Abstract did not contain calculable risk estimates such as ORs (Odds Ratios) |
| Injection drug use among homeless adults with severe mental illness | DOI: 10.2105/ajph.87.5.854 | Abstract did not contain calculable risk estimates such as ORs (Odds Ratios) |

**Table – Data extraction information**

| **STUDY** | **YEAR** | **NDD Types** | **Country** | **Study Design** | **Population** | **Period** | **Age** | **Title** | **Name of data extractors** | **Date of data extraction** | **Confirmation that the study was eligible to be included in the review** |
| --- | --- | --- | --- | --- | --- | --- | --- | --- | --- | --- | --- |
| Jutkowitz et al. | 2022 | ADRD | United States | Cross-sectional study | 6580126 | 2018 | All ages | Prevalence of Alzheimer's disease and related dementias among veterans experiencing housing insecurity | PF | 24-Jul-24 | Confirmed |
| Roncarati JS et al. | 2024 | ADRD | United States | Cohort study | 88811 | 2011-2019 | 63.5 | Risk of dementia among veterans experiencing homelessness and housing instability | PF | 24-Jul-24 | Confirmed |
| Keigher et al. | 1992 | Dementia | United States | Cross-sectional study | 475 | 1987-1988 | ≥ 60 | Housing emergencies and the etiology of homelessness among the urban elderly | PF | 24-Jul-24 | Confirmed |
| Jutkowitz et al. | 2019 | Dementia | United States | Cross-sectional study | 114013 | 2010-2017 | 54.53333 | Homeless Veterans in Nursing Homes: Care for Complex Medical, Substance Use, and Social Needs | PF | 24-Jul-24 | Confirmed |
| Ye et al. | 2019 | Memory loss | United States | Cross-sectional study | 64 | 2016-2018 | ≥ 50 | Health Care Needs of Homeless Older Adults: Examining the Needs of a Senior Center Cohort | PF | 24-Jul-24 | Confirmed |
| Stergiopoulos et al. | 2019 | Cognitive Impairment | Canada | N.A. | 902 | 2009-2011 | ≥ 18 | Housing Stability and Neurocognitive Functioning in Homeless Adults With Mental Illness: A Subgroup Analysis of the At Home/Chez Soi Study | PF | 24-Jul-24 | Confirmed |
| Van Straaten et al. | 2017 | Cognitive Impairment | Netherlands | Cohort study | 513 | 2011 | ≥ 18 | Self-reported care needs of Dutch homeless people with and without a suspected intellectual disability: a 1.5-year follow-up study | PF | 24-Jul-24 | Confirmed |
| Abdollahpour et al. | 2018 | Multiple sclerosis | Iran | Case-control study | 1604 | 2013-2015 | ≥ 13 | Stress-full life events and multiple sclerosis: A population-based incident case-control study | PF | 24-Jul-24 | Confirmed |

*Note: Additional details are available in Table 1 and Figures 2–4 of the main text.*

**An explanation of how missing data were handled**

We excluded studies with missing data on “calculable risk estimates and their 95% CIs,” as stated in the Methods section (“Articles included in this analysis were required to be published in English and to provide calculable risk estimates with their 95% confidence intervals (CIs), such as odds ratios (ORs), relative risks (RRs), hazard ratios (HRs), regression coefficients (βs), or percentage changes (%)”.

If the included articles were with missing data on “descriptive data,” we followed the procedure recorded in the Methods section: “if specific data were not reported in the original sources, they were marked as ‘N/A’. Age ranges were documented as reported; if an age range was not specified, but an average age was available, the average age was noted.
